# How Dendrimers Impact Fibrin Clot Formation, Structure, and Properties

**DOI:** 10.1021/acsomega.4c08120

**Published:** 2024-12-18

**Authors:** Natasha Mina, Vinicius S. Guido, Benedito C. Prezoto, Maria Luiza V. Oliva, Alioscka A. Sousa

**Affiliations:** † Department of Biochemistry, Federal University of São Paulo, São Paulo, SP 04044-020, Brazil; ‡ Laboratory of Pharmacology, Butantan Institute, São Paulo, SP 05503-900, Brazil

## Abstract

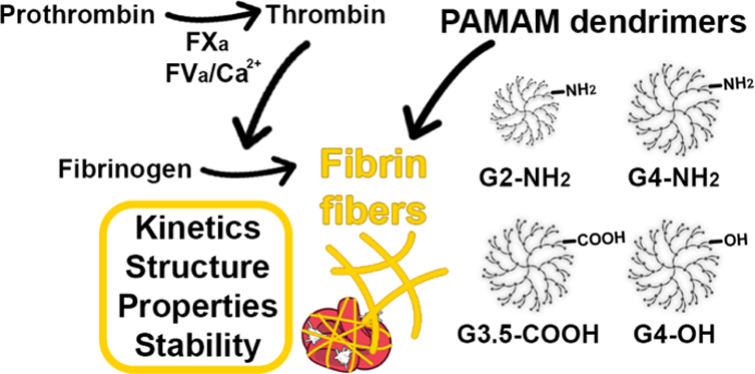

Polyamidoamine (PAMAM) dendrimers, with their unique structural versatility and tunable surface functionalities, have emerged as promising nanomaterials for a wide range of biomedical applications. However, their in vivo use raises concerns, as unintended interactions between dendrimers and blood components could disrupt the delicate hemostatic balance and lead to serious complications like bleeding or thrombosis. In this study, we explored the impact of low-generation PAMAM dendrimers on the kinetics of fibrin clot formation, along with their influence on the structure, properties, and resistance to lysis of the resulting clots. For this purpose, we employed a multilevel characterization approach using purified fibrinogen, human plasma, and whole blood to assess the effects of four dendrimer types: G2-NH_2_, G4-NH_2_, G3.5-COOH, and G4-OH. Among the main findings, both G2-NH_2_ and G4-NH_2_ significantly impaired thrombin generation and delayed clot formation, with G4-NH_2_ also promoting fibrin aggregation, increasing clot permeability, and accelerating clot lysis. When present at high concentrations, G4-OH also affected critical clotting parameters, delaying thrombin generation and prolonging clotting time. Notably, the prolongation of clotting time by G4-OH was evident in both human plasma and whole blood. Interestingly, G3.5-COOH showed potential as a safer option since it induced minimal alterations across most tested metrics. These results will be important for guiding the rational design of dendrimers and identifying safe concentrations for future clinical applications.

## 1 Introduction

Dendrimers are highly branched, three-dimensional polymeric macromolecules. Their tunable architecture allows for precise control over size, shape, and surface functionality.^1,2^ Due to these characteristics, dendrimers are utilized in various biomedical applications, including drug delivery and imaging.^3−5^ Low-generation dendrimers, with molecular weights up to ∼30,000 Da and sizes of up to ∼6 nm, are employed in the majority of preclinical biomedical investigations.^3,4,6−9^

Recently, dendrimers have advanced into human clinical trials.^10−14^ Promising applications include the use of drug-conjugated PEGylated polylysine dendrimers for cancer treatment.^12^ In addition, G4-OH dendrimers conjugated to N-acetyl cysteine, an anti-inflammatory and antioxidant agent, have been assessed for treating hospitalized patients with severe COVID-19.^11,13^

As dendrimers become more prevalent in clinical trials, a rigorous preclinical safety evaluation is essential to identify potential toxicity concerns prior to patient administration.^15−20^ In particular, unintended interactions between dendrimers and blood components could disrupt the delicate hemostatic balance and lead to life-threatening complications.

**Figure 1:**
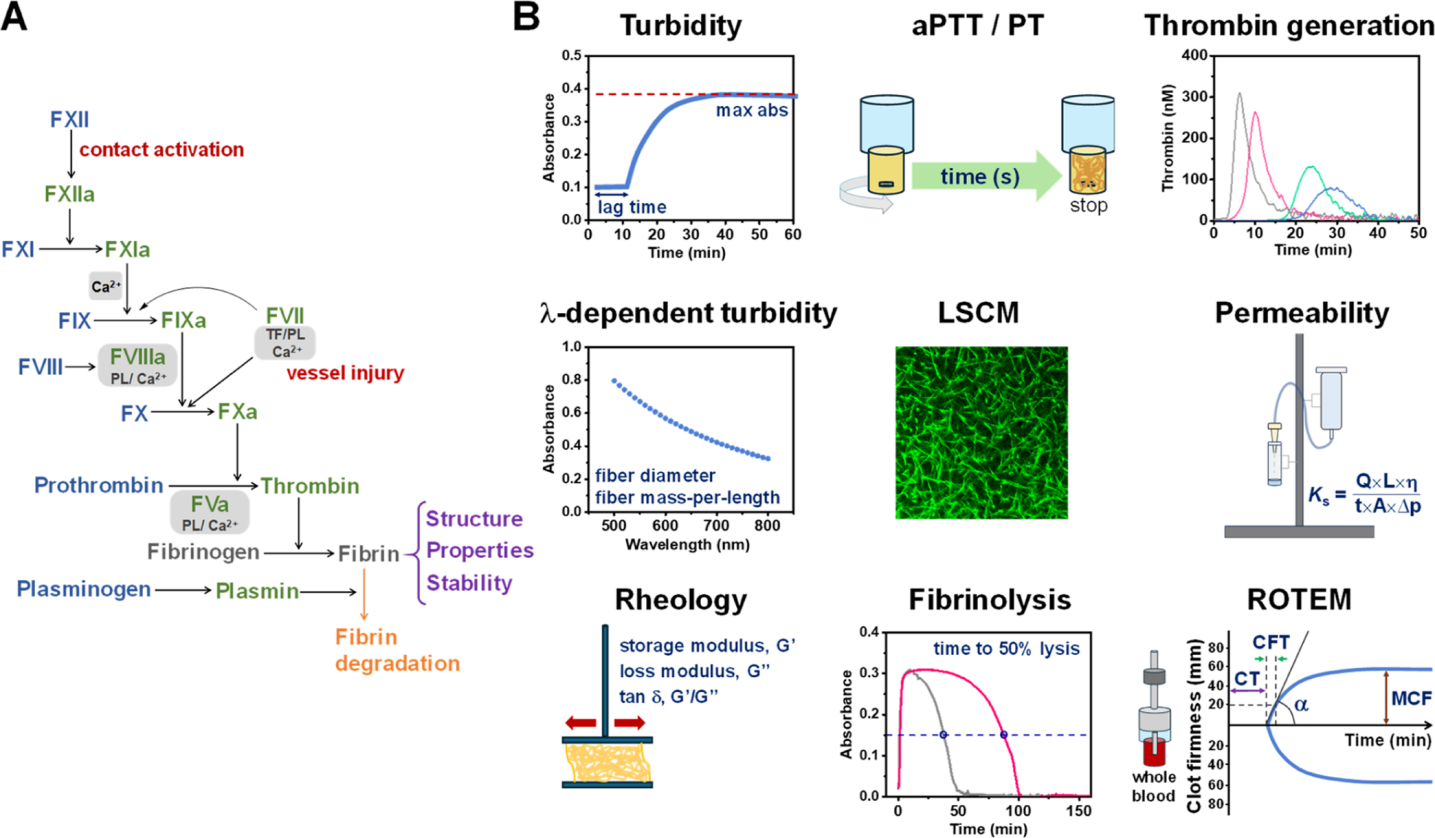
Study overview. (A) Simplified schematic of the plasma coagulation cascade. (B) Illustration of experimental techniques used to assess the impact of dendrimers on thrombin generation and fibrin clot formation.

In normal blood clotting, the exposure of tissue factor (TF) within the lumen of injured blood vessels triggers the coagulation cascade that ultimately leads to the thrombin-mediated proteolytic cleavage of fibrinogen into insoluble fibrin (Figure 1A).^21^ The resultant fibrin network, in conjunction with blood cells, forms the thrombus (or blood clot) that seals the injured blood vessel and stops bleeding. Subsequently, the fibrinolytic system acts over time to dissolve the formed thrombus and allow the restoration of normal blood flow. Blood clotting can also be triggered by contact with exogenous anionic surfaces, such as nanomaterials or biomaterials.^22−24^ In this scenario, the coagulation cascade is initiated by activating the plasma contact system, a process that involves conversion of the factor XII (FXII) zymogen into the proteolytically active form FXIIa (Figure 1A).^21,25^ We further emphasize that the formation of fibrin clots having proper structural and biomechanical properties—including fiber thickness, network density/porosity, permeability, and stiffness/plasticity—is crucial for ensuring effective hemostasis and wound healing.^26−28^

Previous studies have explored the interactions and potential effects of dendrimers on blood cells,^29−33^ as well as their impact on the contact, coagulation, and fibrinolytic systems.^34−39^ It has been found that dendrimers can modulate the activity of coagulation enzymes and cause alterations to key hemostatic parameters such as thrombin generation and clotting time, depending on dendrimer generation, concentration, and surface chemistry. However, there has been limited attention given to the integrated impact of dendrimers on the formation, structure, properties, and stability of fibrin clots. Addressing this research gap is crucial, since abnormal clot formation can pose a significant risk for individuals.^26,40−42^ Indeed, various diseases and pathologies can result in altered clotting kinetics and impaired clot structure, leading to an increased tendency for bleeding or thrombosis. For example, hematologic conditions like hemophilia A and FXI deficiency increase susceptibility to bleeding,^41,43^ whereas individuals with cardiovascular disease, type-2 diabetes, and cirrhosis face an elevated risk of thrombosis.^42,44−47^ Additionally, a recent study explored the clot formation process on blood-contacting medical devices, revealing the influence of material wettability on fibrin network structure and stability, and the potential implications for material-induced thrombosis.^48^

We have recently explored how anionic ultrasmall gold nanoparticles (usGNPs) with a hydrodynamic diameter of about 3.5 nm—similar to polyamidoamine (PAMAM) G3/G4 generation dendrimers—interfere with the coagulation system and impact fibrin clot formation.^49−51^ These ultrasmall particles interacted with fibrinogen, delayed the kinetics of clot formation, and disrupted the normal architecture of the fibrin network, leading to larger clot pore sizes and increased clot permeability to liquid. These previous findings imply the potential for similar adverse effects of dendrimers on the fibrin clot formation process.

Based on the above considerations, herein we employ an array of analytical techniques to explore the impact of dendrimers on the formation, structure, properties, and stability of fibrin clots. As relevant models, we utilize a series of low-generation PAMAM dendrimers, including cationic (G2-NH_2_ and G4-NH_2_) and anionic (G3.5-COOH) dendrimers, as well as the clinically relevant hydroxyl-terminated G4 dendrimer (G4-OH).

## 2 Materials and Methods

### 2.1 Reagents

PAMAM dendrimers were from Sigma-Aldrich (São Paulo, Brazil). Human fibrinogen (plasminogen-depleted) was from Enzyme Research (South Bend, IN). Human α-thrombin, tPA, and corn trypsin inhibitor (CTI) were from Innovative Research (Novi, MI). Actin FS, Thromborel S, and Dade Innovin were from Siemens Healthineers (Erlangen, Germany). Arachidonic acid was from Sigma-Aldrich (São Paulo, SP, Brazil). The substrate Z-Gly-Gly-Arg-AMC was from Bachem (Torrance, CA). AlexaFluor 488-conjugated fibrinogen was from Merck (São Paulo, Brazil). The following buffer solutions were prepared before each experiment according to standard protocols: TBS (100 mM Tris–HCl, 150 mM NaCl, 0.01% tween 20, pH 7.4), HBS (10 mM HEPES, 150 mM NaCl, 0.1% PEG8000, pH 7.4), and HEPES–Tyrode (5 mM HEPES, 137 mM NaCl, 2.9 mM KCl, 12 mM Na_2_HPO_4_, 5 mM C_6_H_12_O_6_, 1 mM CaCl_2_, 1 mM MgCl_2_, pH 7.4).

### 2.2 Blood Collection

Blood was drawn from five volunteers following approval from the institution’s Research Ethics Committee. Collection was done in BD Vacutainer tubes (BD Bioscience, São Paulo, Brazil) containing sodium citrate as an anticoagulant. Platelet-poor plasma was prepared from the anticoagulated blood following standard protocols.^52^ Aliquots of plasma were stored at −80 °C until use. Before each experiment, plasma samples were thawed in a 37 °C water bath and used immediately, with any remaining volume discarded. Additionally, fresh citrated whole blood from up to seven individual donors was used directly in ROTEM experiments, as detailed in Section 2.12.

### 2.3 Fibrin Clot Formation through Optical Turbidimetry

Purified fibrinogen (4 μM, or 1.4 g/L) in TBS buffer was incubated with dendrimers (1, 10, and 50 μM) for 15 min at room temperature (RT) in a 96-well plate, followed by the addition of thrombin (0.2 NIH/mL) to initiate clotting. Clot formation was monitored at 37 °C over time through turbidity readings at 350 nm using a VersaMax microplate reader (Molecular Devices). From the progress curves, the time to 5% clotting was determined with the “Clotting_or_HaloCL” Shiny app.^53^ The impact of dendrimers on the kinetics of clot formation was further evaluated in human plasma. For this purpose, citrated plasma was diluted 1:3 in HBS and incubated with dendrimers (1, 10, and 50 μM) for 30 min at RT. Clot formation was initiated by adding thrombin (0.2 NIH/mL) or the aPTT reagent (25% v/v) with CaCl_2_ (10 mM), and then monitored over time by measuring turbidity at 350 nm. All concentrations were final after mixing.

### 2.4 aPTT and PT Assays

For the aPTT assay, citrated human plasma was incubated with and without dendrimers (1, 10, and 50 μM) in HBS buffer for 10 min at 37 °C. Following this, samples were incubated with Actin FS (25% v/v) for 1 min and then recalcified (10 mM CaCl_2_). The time to clot formation was monitored using a Dade Behring BFT-II semiautomated coagulation analyzer (Siemens Healthineers). For the PT assay, a similar procedure was followed, except that Thromborel S (50% v/v) was used to activate the coagulation cascade. All concentrations and volume fractions were final after mixing.

### 2.5 Thrombin Generation Assay (TGA)

Citrated human plasma, containing CTI (10 μM) to prevent contact activation of FXII, was incubated with and without dendrimers (1, 10 and/or 50 μM) for 30 min at 37 °C in a 96-well plate. A low concentration of recombinant human TF (Innovin reagent; 1 pM) was used to trigger the coagulation cascade. Then, a premixed solution containing CaCl_2_ (10 mM) and the thrombin-specific Z-Gly-Gly-Arg-AMC fluorogenic substrate (420 μM) was added to the well plate immediately before the start of data collection. The total volume in each well was 120 μL, with the plasma volume corresponding to 80 μL. All concentrations were final after mixing. Additional experiments were conducted similarly, except that Actin FS (4.2% v/v) was used to trigger the coagulation cascade in citrated human plasma without CTI. Data collection was carried out on a FlexStation 3 microplate reader (Molecular Devices) using excitation and emission wavelengths of 390 and 460 nm, respectively. Thrombin generation curves were calculated with the “ThrombinCL” Shiny app.^53^

### 2.6 Clot Lysis

Citrated human plasma diluted 1:3 in HBS buffer was incubated with and without dendrimers (1, 10, and 50 μM) and tPA (1.0 nM) for 30 min at RT in a 96-well plate. The plasma samples were then treated with thrombin (0.4 NIH/mL) and CaCl_2_ (10 mM) to initiate clotting. All concentrations were final after mixing. The amount of tPA was adjusted so that clot lysis began only after reaching the same maximum absorbance as in the absence of tPA, indicative of a fully formed clot. Clot formation and lysis were monitored at 37 °C through turbidity readings at 350 nm using a microplate reader. Time to 50% lysis was determined using the “ClotLysisCL” Shiny app.^53^

### 2.7 Wavelength-Dependent Turbidimetry

Fibrinogen (4 μM) in TBS buffer was loaded into 1 cm wide cuvettes and incubated with and without dendrimers (10 and 50 μM) at RT for 30 min. This was followed by the addition of thrombin (0.4 NIH/mL) to initiate clotting. In another set of experiments, human plasma diluted 1:6 in HBS was incubated with and without dendrimers (1, 10, and 50 μM) at RT for 30 min, followed by the addition of thrombin (0.4 NIH/mL) and CaCl_2_ (10 mM) to initiate clotting. Both sets of samples were incubated for an additional 1.5 h to stabilize and consolidate the clots. Absorbance readings were performed using a Shimadzu UV–1800 spectrophotometer, with measurements covering the range from 500 to 800 nm. The determination of fibrin fiber diameter was performed using the corrected Yeromonahos approach, implemented in an Excel spreadsheet provided by Belcher et al.^54^ This approach offers the most accurate diameter values, with <20% error within the range of ∼130 to 260 nm. Values of mass-per-length (MPL) ratio were calculated using the Carr-Hermans approach, also implemented in an Excel spreadsheet provided by the same authors.^55^ The number of protofibrils per fiber was then calculated by dividing the obtained MPL values by the MPL of a single protofibril (1.44 × 10^11^ Da/cm).^56^ This approach shows <20% error for fibers up to 200 nm in diameter. It is important to note that these error estimates are based on fibers being sufficiently longer than the wavelength of light.

### 2.8 Laser Scanning Confocal Microscopy

Citrated human plasma diluted 1:3 in HBS buffer was spiked with AlexaFluor 488-conjugated fibrinogen (50 nM), making up approximately 0.8–1.6% of the total fibrinogen. The plasma samples were transferred to a 35 mm glass-bottom dish and incubated with and without dendrimers (10 and 50 μM) at RT for 30 min. Subsequently, clot formation was triggered by adding thrombin (0.8 NIH/mL) and CaCl_2_ (10 mM), and samples were further incubated at RT in a humidity chamber for 2 h prior to imaging. All concentrations were final after mixing. Clots were imaged using a Zeiss LSM 780 confocal microscope. The confocal data were collected from two independent experiments, each performed in technical duplicate. Approximately 30 optical sections were captured at 0.4 μm intervals using a × 40-magnification oil objective. Control samples lacking thrombin and Ca^2+^ were prepared under identical conditions to evaluate if dendrimers induced fibrinogen aggregation independently.

### 2.9 Clot Permeability

Undiluted citrated human plasma was incubated with and without dendrimers (50 and 150 μM) at RT for 30 min. Next, thrombin (0.8 NIH/mL) and CaCl_2_ (10 mM) were added to initiate clotting, and a small volume of the mixture (100 μL) was immediately transferred (before clot formation) to a 4.5 cm plastic pipet tip. The samples were kept in a humidity chamber at RT for 2 h to allow full clot formation and stabilization. After this, the plastic tip was joined to a syringe filled with HBS buffer through a silicon tube. The clot was washed with buffer for 1.5 h before sample collection began. Permeation measurements were performed by collecting the volume of buffer passing through the clot under a constant 4 cm pressure drop. Clot permeability was calculated using Darcy’s formula:
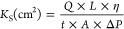
1where *Q* (cm^3^) is the volume of buffer collected at a given time *t*, *L* is the length of the clot (1.7 cm), η is the liquid viscosity (10^–7^ dyne·s·cm^–2^), *A* is the cross-sectional area of the clot (0.071 cm^2^), and Δ*P* is the pressure drop (0.04900 dyn cm^–2^).

### 2.10 Clot Rheometry

Citrated human plasma diluted 1:2 in HBS buffer was incubated with and without dendrimers (10 and 50 μM) for 30 min at 37 °C. The samples were then transferred to a rheometer (Anton-Paar) and clotting was initiated by adding thrombin (0.4 NIH/mL) and CaCl_2_ (10 mM). All concentrations were final after mixing. The storage modulus (*G*′) and loss modulus (*G*′’) during clot formation were monitored over time in oscillatory mode, using a frequency of 1 Hz and a strain (γ) of 1% for 1500 s.

### 2.11 Hemolysis and Platelet Aggregation

The general experimental procedure for investigating the influence of dendrimers on red blood cell (RBC) hemolysis and platelet aggregation is outlined in the Supporting Information, and it has also been detailed in previous reports.^57^

### 2.12 Rotational Thromboelastometry

Citrated human whole blood samples (300 μL) were incubated with G3.5-COOH and G4-OH at 37 °C for 30 min. Blood from three individual donors was used to obtain data for G3.5-COOH at a concentration of 50 μM, while blood from four donors was used for G4-OH at concentrations of 1, 10, and 50 μM. Control samples contained no dendrimers. Coagulation was initiated by adding Actin FS (15% v/v) with CaCl_2_ (10 mM) or Thromborel S (15% v/v) with CaCl_2_ (10 mM). All concentrations reflect final values after mixing. Samples were analyzed using a four-channel computerized ROTEM system (Pentapharm, Germany).

### 2.13 Statistical Analysis

Data are presented as mean ± SD. Statistical significance between two groups was assessed using Student’s *t* test, while comparisons among multiple groups were performed using one-way ANOVA followed by Tukey’s posthoc test. A *p*-value <0.05 was considered statistically significant.

## 3 Results and Discussion

We adopted a multilevel characterization approach to assess the potential impact of dendrimers on fibrin clot formation and blood clotting, utilizing samples of purified fibrinogen, human plasma, and human whole blood. The various methods employed are summarized in Figure 1B. Notably, the chosen set of techniques is commonly used to understand how various health conditions—such as cirrhosis, cardiovascular diseases, clotting factor deficiencies, infections, sepsis, and even exposure to particulate air pollution—influence multiple aspects of blood clotting.^42−44,46,58−61^

To establish a reasonable range of dendrimer concentrations, we took into account the peak plasma concentration of 4 μM for PAMAM G4-OH-based dendrimers reported in a recent human clinical trial.^11^ The concentrations we used in our experiments primarily included 1 and 10 μM, which are similar to the peak levels found in vivo, and 50 μM, which is roughly 10 times higher than the peak level.

Before beginning, we employed optical turbidimetry to ensure that the dendrimers alone did not induce fibrinogen aggregation in samples of purified fibrinogen and citrated human plasma (Supporting Figure S1A). This outcome differs from a previous study in which a high-generation cationic G7 PAMAM dendrimer was found to cause rapid and extensive fibrinogen aggregation in a thrombin-independent manner.^38^ Additionally, we confirmed that none of the dendrimers activated the coagulation cascade at the level of FXII (Supporting Figure S1B). The observation that anionic G3.5-COOH did not activate FXII contrasts with our previous findings involving anionic usGNPs of a comparable size, which were found to interact with and convert FXII into FXIIa.^50^ Overall, these preliminary results indicated that the dendrimers themselves did not produce discernible changes in anticoagulated (citrated) human plasma.

### 3.1 Dynamics of Fibrin Clot Formation

**Figure 2:**
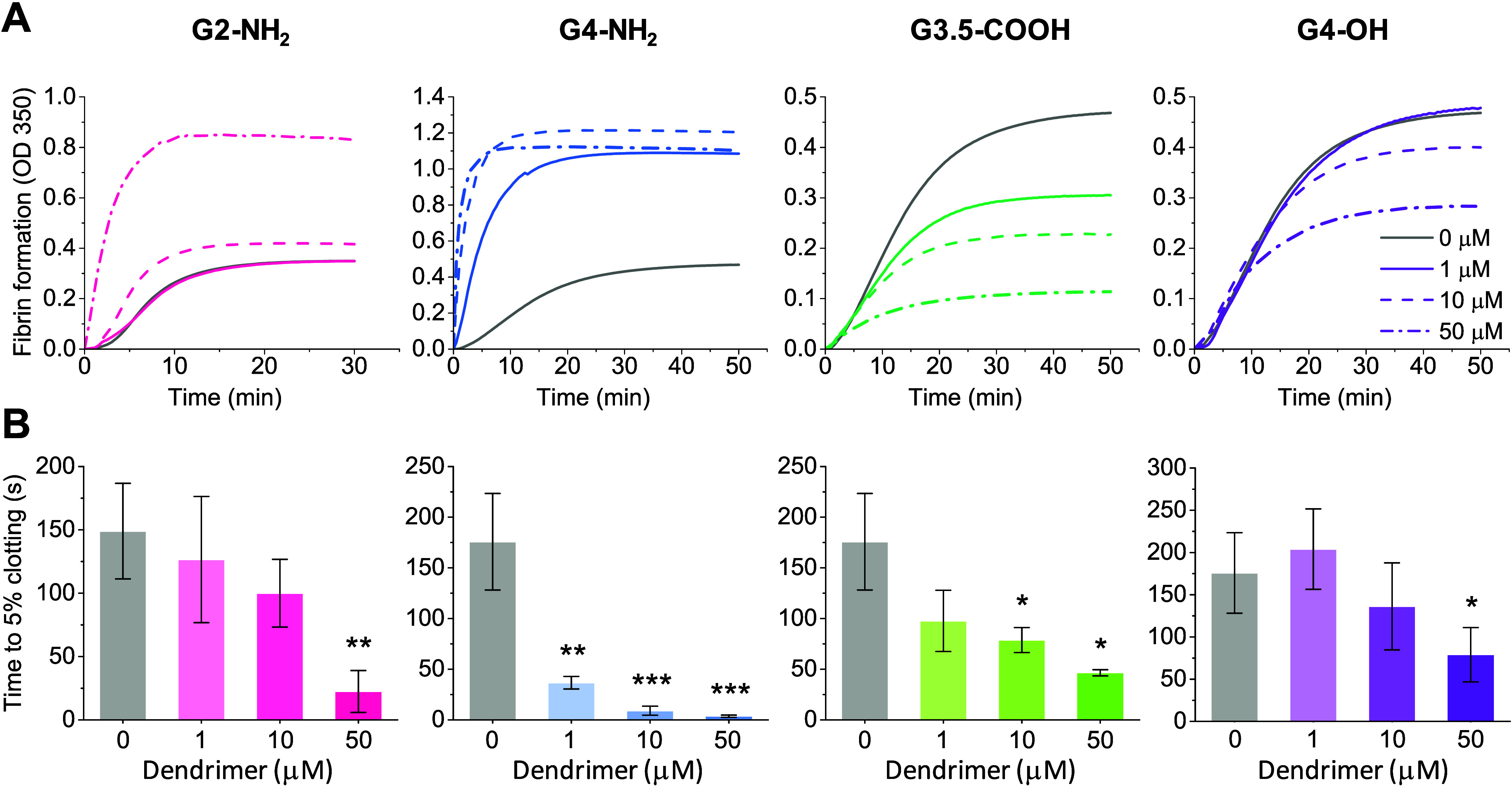
Influence of dendrimers on the dynamics of fibrinogen clotting evaluated through optical turbidimetry. Fibrinogen (4 μM) was incubated with dendrimers in TBS buffer at the indicated concentrations for 15 min, followed by the addition of thrombin (0.2 NIH/mL). (A) Progress curves of fibrin clot formation. Curves represent the average of three measurements. The legend under G4-OH applies to all panels. (B) Time to 5% clotting calculated from progress curves. Data are reported as mean ± SD (*n* = 3), with *p < 0.05, **p < 0.01, and ***p < 0.001 relative to vehicle control.

We employed optical turbidimetry to examine the impact of dendrimers on the dynamics of fibrin clot formation. This method relies on the light scattering caused by fibrin fibers, leading to increased turbidity in the solution over time. Initially, we monitored the clot formation process in a simplified system consisting of purified fibrinogen, using thrombin to initiate clotting. From the progress curves (Figure 2A), the time to 5% clotting was extracted and used as a measure of clotting time (CT) (Figure 2B). It can be seen that G4-NH_2_ drastically accelerated clot formation, with G2-NH_2_ exhibiting a similar but less pronounced effect. As a control, we confirmed that G4-NH_2_ did not significantly increase thrombin activity relative to the other dendrimers, ruling it out as a factor for the trends observed in Figure 2 (Supporting Figure S2). These findings are consistent with those previously reported by Aisina et al.^37^ The pronounced acceleration of clotting dynamics by G4-NH_2_ is likely the result of fibrin aggregation, as discussed below. G3.5-COOH also shortened CT in a dose-dependent manner; however, unlike G4-NH_2_, this effect was linked to a decrease in maximum turbidity. We note that the outcome for G3.5-COOH contrasts sharply with that for anionic usGNPs of similar size, which significantly delayed fibrinogen clotting.^49^ Although the reason for this difference is not yet understood, we note that dendrimers are more flexible structures than usGNPs, which may influence their modes of interaction and binding strength toward fibrinogen.

**Figure 3:**
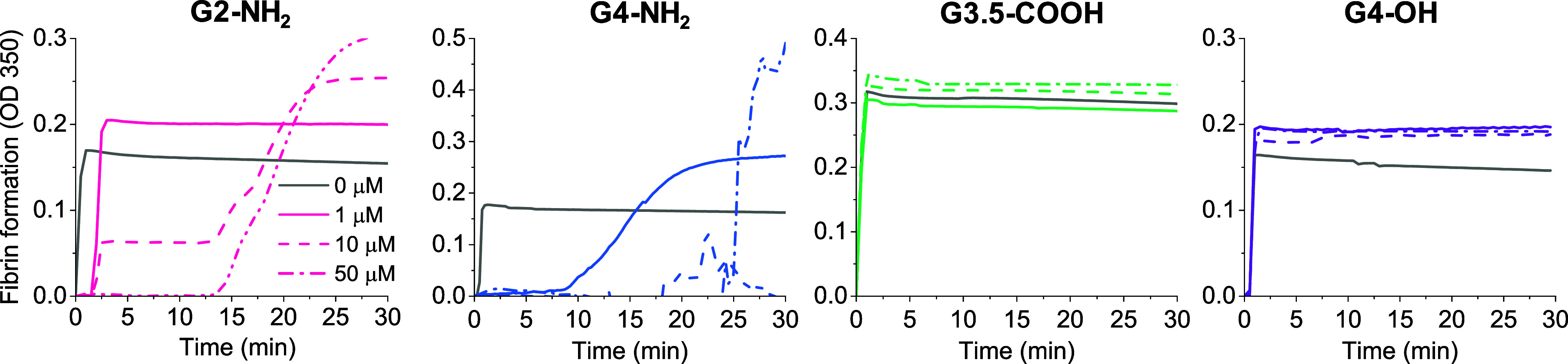
Influence of dendrimers on plasma clot formation evaluated through optical turbidimetry. Citrated human plasma was diluted 1:3 in HBS and incubated with dendrimers at the indicated concentrations for 30 min. Clot formation was initiated using Actin FS and Ca^2+^ (10 mM). Shown are progress curves of fibrin clot formation. Curves represent the average of three measurements. The legend under G2-NH_2_ applies to all panels.

Next, we assessed the impact of dendrimers in the more physiological setting of blood plasma. First, we incubated citrated human plasma with dendrimers and added thrombin to trigger clotting. It can be seen that none of the dendrimers had a noticeable impact on clot formation kinetics; however, maximum turbidity increased, particularly for G2-NH_2_ and G4-NH_2_ (Supporting Figure S3). In separate experiments, we used the activated partial thromboplastin time (aPTT) reagent, Actin FS, which contains phospholipids and ellagic acid, to initiate the coagulation cascade at the level of FXII. Under these conditions, G3.5-COOH and G4-OH did not alter the progress curves, whereas both G2-NH_2_ and G4-NH_2_ significantly delayed the clotting process (Figure 3), with lag times extending to ∼2–25 min depending on the dendrimer type and concentration.

**Figure 4:**
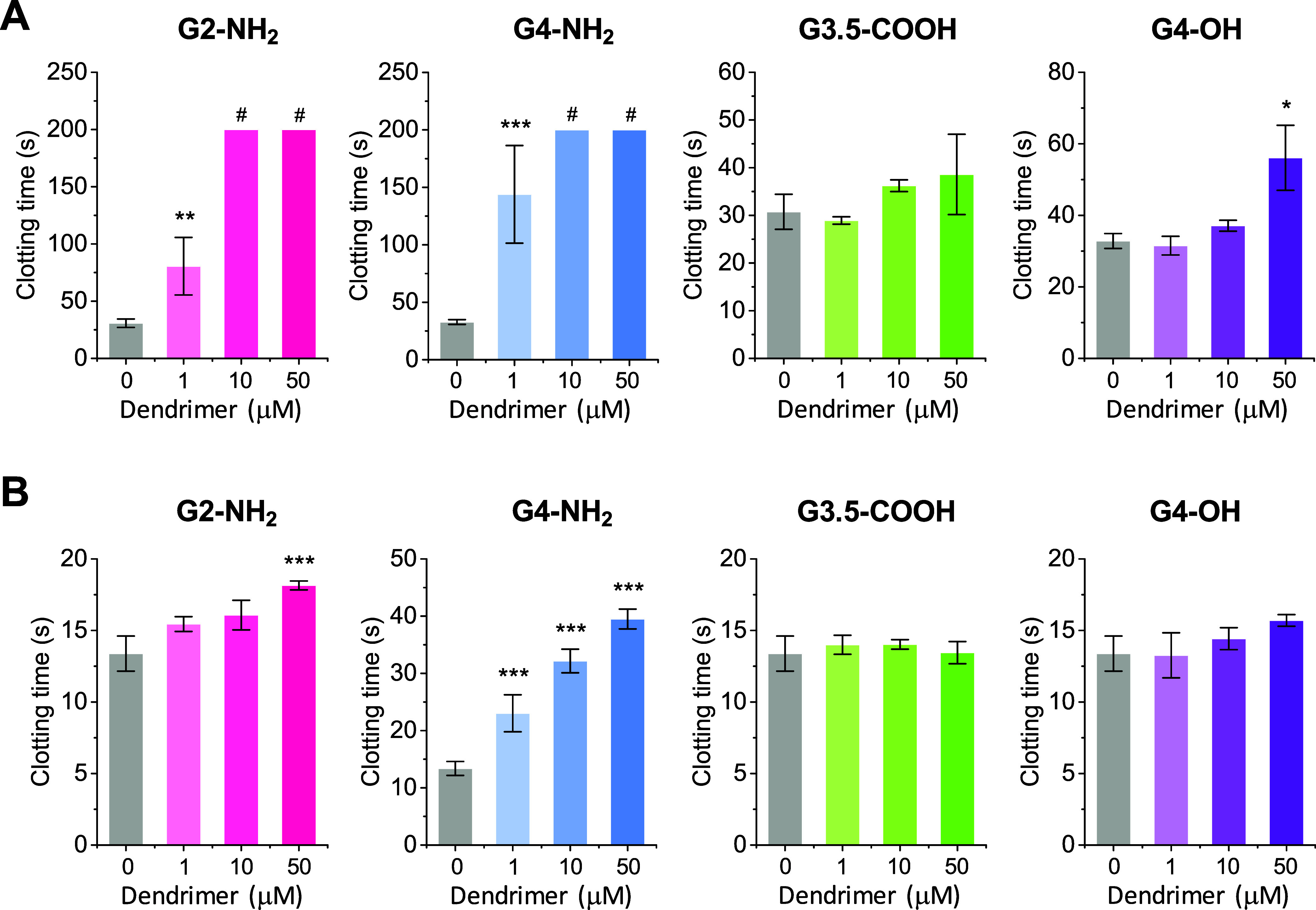
Influence of dendrimers on the time to clot formation in human plasma assayed through the (A) aPTT and (B) PT standardized tests. Citrated human plasma was diluted 1:4 in HBS and incubated with dendrimers at the indicated concentrations. Clot formation was initiated using (A) Actin FS with Ca^2+^ (10 mM) or (B) Thromborel S. Data are reported as mean ± SD (*n* = 4), with *p < 0.05, **p < 0.01, and ***p < 0.001 relative to vehicle control. Symbol # designate lack of clot formation up to the maximum recorded assay time.

To complement the above studies, we investigated the impact of dendrimers on clot formation using the standardized aPTT and prothrombin time (PT) assays, which probe the “intrinsic” and “extrinsic” coagulation pathways, respectively.^62^ For this purpose, plasma samples containing dendrimers were activated at the level of FXII and TF/factor VIIa using the Actin FS and Thromborel S reagents, respectively. The corresponding CT was then recorded using a mechanical coagulometer. Results for the aPTT assay showed that G3.5-COOH did not change CT beyond the clinically accepted range, while G4-OH increased CT to 55 s when present at a concentration of 50 μM (Figure 4A). On the other hand, both cationic dendrimers increased CT even at a lower concentration of 1 μM, and at the highest concentrations, the samples failed to clot within 200 s of measurement (Figure 4A). Results from the PT assay indicated that G2-NH_2_, and particularly G4-NH_2_, prolonged CT, whereas G3.5-COOH and G4-OH showed no effect (Figure 4B). Our findings for G4-NH_2_ are consistent with those reported by Markowicz-Piasecka et al.^34^

In sum, the above results demonstrate that dendrimers can have varying effects on the dynamics of clot formation depending on the investigated system (purified fibrinogen vs human plasma), dendrimer type and concentration, and measurement method. Most significantly, experiments conducted in human plasma demonstrated that G2-NH_2_ and G4-NH_2_ significantly delayed the onset of clot formation. However, this effect was observed only when clotting was initiated at the level of FXII or TF, not when triggered by thrombin. This suggests that both dendrimers interfere with coagulation reactions upstream of thrombin, a hypothesis further corroborated in Section 3.3. Conversely, both G3.5-COOH and G4-OH did not affect the onset of clot formation in human plasma, except for G4-OH at high concentrations in the aPTT assay.

### 3.2 Maximum Turbidity of Fibrin Clots

The maximum absorbance observed in turbidimetry traces is influenced by several factors, including fibrinogen concentration, the ultrastructure of fibrin fibers (fiber thickness and mass-per-length ratio), and fibrin aggregation. In samples of purified fibrinogen (Figure 2A), both G3.5-COOH and G4-OH reduced the maximum turbidity, with G3.5-COOH showing a pronounced effect (see Section 3.4 for further analysis). In contrast, G2-NH_2_ and G4-NH_2_ increased turbidity, with G4-NH_2_ showing a particularly significant change. For both G2-NH_2_ and G4-NH_2_, the large increase in maximum turbidity combined with the significant acceleration of CT suggests that these dendrimers actually induced fibrin aggregation in the presence of thrombin. In samples of human plasma activated with the aPTT reagent, both G2-NH_2_ and G4-NH_2_ appeared to induce fibrin aggregation, as evidenced by the erratic increase in absorbance over time (Figure 3).

### 3.3 Dynamics of Thrombin Generation

The time course of thrombin generation (TG) in human plasma is a key factor influencing fibrin polymerization and the ensuing clot structure.^63^ Hence, dendrimers might affect clot formation and structure by altering TG dynamics. To investigate this, we employed the thrombin generation assay (TGA) to measure real-time TG in the presence of dendrimers.^64,65^

**Figure 5:**
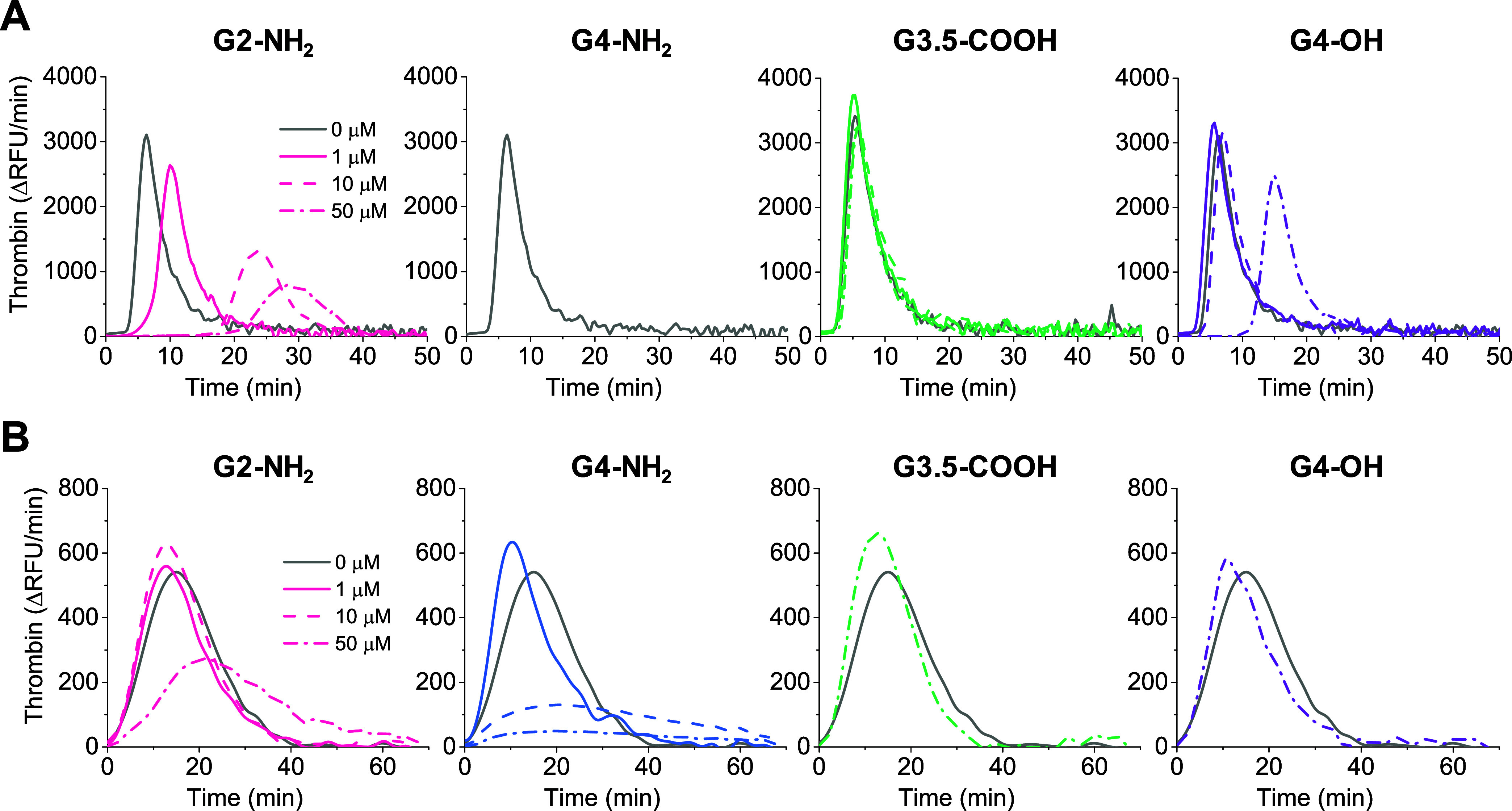
Influence of dendrimers on real-time thrombin generation in human plasma. Shown are thrombin generation curves, representing the average of 4–6 measurements. Citrated human plasma was incubated with dendrimers at the indicated concentrations for 30 min. Clotting was initiated using (A) Actin FS with Ca^2+^ (10 mM) or (B) recombinant TF (Innovin) with Ca^2+^ (10 mM). No thrombin generation was detected in plasma incubated with G4-NH_2_ and treated with Actin FS. Corresponding thrombin generation parameters are included in Supporting Figures S4 and S5. The legend under G2-NH_2_ applies to all panels.

First, we used Actin FS to trigger the intrinsic coagulation pathway through FXII activation. TGA curves are shown in Figure 5A, while corresponding TG parameters derived from TGA curves are displayed in Supporting Figure S4. It can be seen that G2-NH_2_ significantly impaired TG, specifically prolonging the lag time and time to peak, while decreasing the peak thrombin concentration and the endogenous thrombin potential (area under the curve). The effect of G4-NH_2_ was even more drastic, completely inhibiting TG. In contrast, G3.5-COOH had no influence on TG, while G4-OH surprisingly prolonged lag time and time to peak, although only at the highest concentration of 50 μM. Next, we used recombinant TF (Innovin) to trigger the coagulation cascade. A low concentration of TF was employed to probe the intrinsic coagulation pathway through the thrombin-mediated activation of factor XI.^65^ TGA curves and derived TG parameters are shown in Figure 5B and Supporting Figure S5, respectively. It can be seen that G2-NH_2_ and G4-NH_2_ reduced peak thrombin concentration and endogenous thrombin potential, with the larger G4-NH_2_ having a more pronounced effect. In contrast, G3.5-COOH and G4-OH only mildly affected TG dynamics up to a concentration of 50 μM. These results are partly consistent with those previously reported by Aisina et al.^37^

Taken together, the above findings indicate that both G2-NH_2_ and G4-NH_2_ significantly interfere with normal TG. This readily accounts for the observed delay in the onset of clot formation as determined by optical turbidimetry (Figure 3) and the aPTT and PT assays (Figure 4). The mechanism driving this phenomenon may be partly related to inhibition of factor X activity.^34^ For G4-OH, the observed impairment of TG with Actin FS, but not recombinant TF, implies interference within the contact pathway; however, a detailed mechanistic investigation was beyond our scope. Nevertheless, the TGA results for G4-OH are partly consistent with the findings from the aPTT and PT assays, which showed a prolonged CT in the aPTT but not in the PT test (Figure 4).

For the experiments described in sections 3.4 through 3.8, clotting in human plasma was initiated using thrombin and CaCl_2_. This allowed us to assess the effects of dendrimers on fibrin clot structure, properties, and stability without the confounding influence of TG rates.

### 3.4 Fibrin Fiber Diameter and Protofibril Number per Fiber

We applied wavelength-dependent turbidity as a powerful technique for characterizing the ultrastructural properties of fibrin fibers under wet conditions. This method provides parameters such as average fiber diameter, mass-per-length ratio, number of protofibrils per fiber, and protofibril distance within a fiber.^56^ Several models are available in the literature to fit turbidimetry data and extract these metrics. Recently, Hudson and colleagues evaluated the available models against scanning electron microscopy and superresolution optical imaging to identify the models and conditions that offer the most accurate measurements.^54,55^ This work incorporates these recent advancements. Due to the likelihood of some fibrin aggregation in the presence of G2-NH_2_ and G4-NH_2_ (especially the latter), the following analyses focused solely on G3.5-COOH and G4-OH.

**Figure 6:**
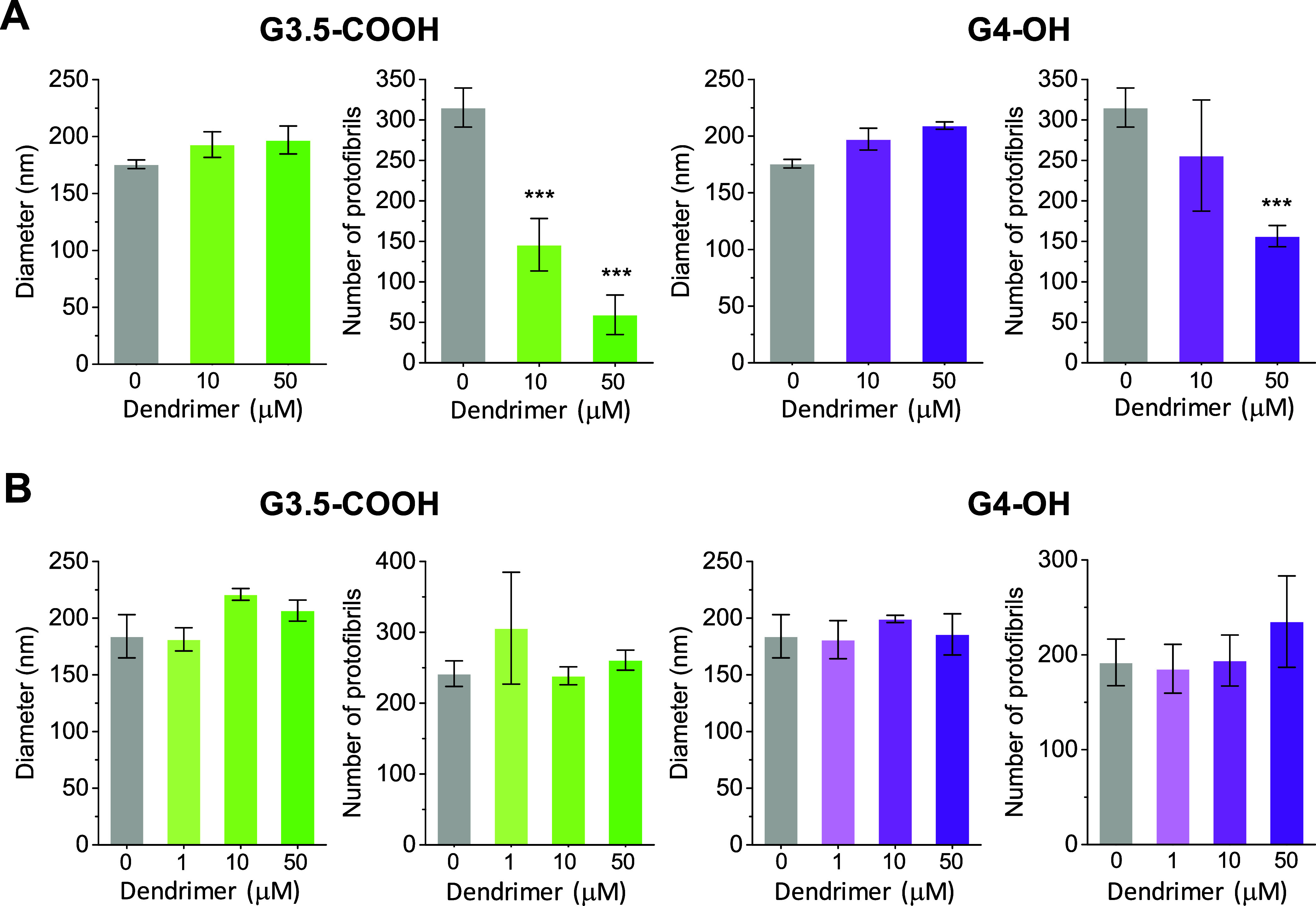
Influence of dendrimers on the ultrastructure of fibrin fibers assessed via wavelength-dependent turbidimetry. The figure presents the average values for the diameter and the number of protofibrils per fibrin fiber within fibrin clots formed from (A) purified fibrinogen and (B) human plasma. Fibrinogen (4 μM) or human plasma (diluted 1:6 in HBS) were incubated with dendrimers for 15 min at the indicated concentrations. Clotting was induced by the addition of thrombin (0.4 NIH/mL) and Ca^2+^ (10 mM). Data are reported as mean ± SD (*n* = 3), with ***p < 0.001 relative to the vehicle control.

We began by assessing the impact of G3.5-COOH and G4-OH on fiber diameter and the number of protofibrils per fiber in samples of purified fibrinogen. For this purpose, fibrinogen was incubated with dendrimers and then treated with thrombin and Ca^2+^ to induce clotting. The results indicated that fiber diameter remained relatively constant at around 175–200 nm in the presence of both dendrimers (Figure 6A). However, both dendrimers significantly reduced the number of protofibrils per fiber, with G3.5-COOH having a more pronounced effect. Next, we conducted similar analyses on samples of human plasma (Figure 6B). Under these conditions, both fiber diameter and protofibril count remained constant for both dendrimers.

### 3.5 Overall Fibrin Network Structure

**Figure 7:**
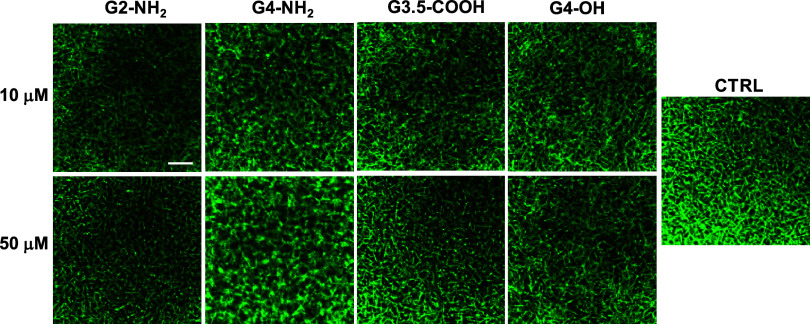
Confocal microscopy characterization of plasma clots. Citrated human plasma spiked with AlexaFluor488-conjugated fibrinogen was diluted 1:3 in HBS and incubated with dendrimers at the indicated concentrations for 30 min. This was followed by the addition of thrombin (0.8 NIH/mL) with Ca^2+^ (10 mM) to trigger clotting. A confocal image of control samples without dendrimers is displayed under the label CTRL. Scale bar, 20 μM.

To further characterize the structural characteristics of the fibrin clot, we employed laser scanning confocal microscopy to visualize the overall clot architecture (Figure 7).^47^ Human plasma was spiked with fluorescently labeled fibrinogen, incubated with dendrimers, and then treated with thrombin and Ca^2+^ to initiate clotting. Except for G4-NH_2_, none of the dendrimers produced notable changes to clot architecture. G4-NH_2_, in contrast, induced local fibrin aggregation, which was especially visible at 50 μM. As a control, none of the dendrimers caused fibrinogen aggregation in the absence of thrombin and Ca^2+^ (not shown).

### 3.6 Clot Permeability

**Figure 8:**
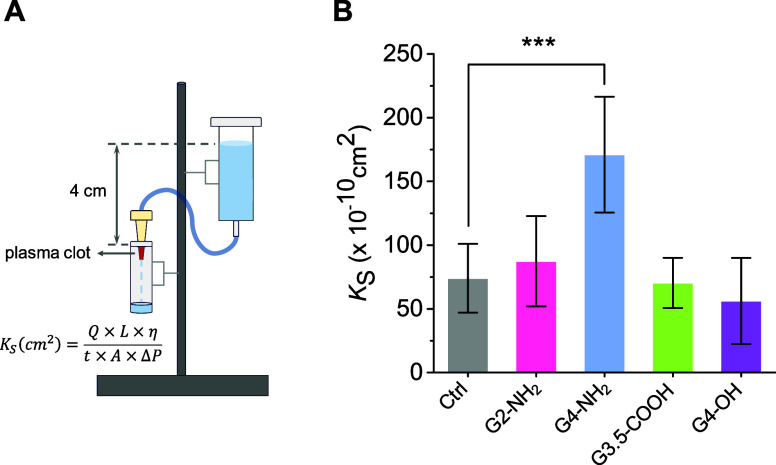
Influence of dendrimers on clot permeability to liquid. (A) Schematic of the clot permeation setup, showing a syringe filled with permeation buffer, a plastic pipet tip containing the fibrin clot (in red color), a connecting plastic tube, and a microtube for collecting the permeated buffer. Clots were formed by adding thrombin (0.8 NIH/mL) with Ca^2+^ (10 mM) to citrated human plasma in the absence or presence of dendrimers (150 μM). (B) Results of clot permeation studies. Darcy’s constant (*K*s) was calculated according to eq 1. Data are reported as mean ± SD (*n* = 5–6), with ***p < 0.001 relative to vehicle control.

Permeability analysis, represented by the Darcy’s constant, quantifies the flow rate of a liquid as it moves through a fibrin mesh.^66^ It provides a key measure of the average pore size and overall density of a fibrin clot. The basic setup of the permeability experiment is depicted in Figure 8A. First, we verified that control measurements of the Darcy’s constant from plasma samples without dendrimers matched those reported in the literature (Figure 8B).^44,61^ In the presence of dendrimers, we found that clot permeability remained unchanged up to concentrations of 50 μM (not shown). Thus, although G4-NH_2_ caused visible structural changes to the clots as depicted in Figure 7, these changes did not translate into altered permeability under the given experimental conditions. To explore this further, we increased the dendrimer concentration by 3-fold. This adjustment led to a significant increase in clot permeability with G4-NH_2_, but not with other dendrimers (Figure 8B), consistent with the trends observed in the confocal results.

### 3.7 Rheological Properties of Plasma Clots

Fibrin clots experience shear stress from blood flow within vessels. Consequently, the biomechanical properties of the fibrin network become essential for its function, with abnormal properties contributing to many thrombotic disorders.^26^

**Figure 9:**
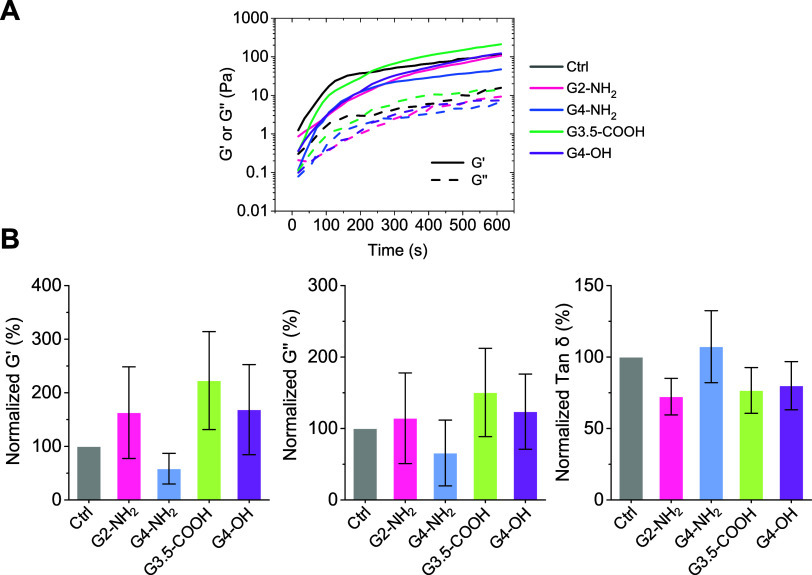
Influence of dendrimers on the rheological properties of fibrin clots. Citrated human plasma was diluted 1:2 in HBS and incubated with dendrimers (50 μM) for 30 min. This was followed by the addition of thrombin (0.4 NIH/mL) with Ca^2+^ (10 mM) to initiate clotting. (A) Storage modulus (*G*′) and loss modulus (*G*′’) curves obtained during plasma clot formation using an oscillatory rheometer. Curves represent the average of 3–6 measurements. (B) Average values for *G*′, *G*′′, and tan δ. Data are reported as mean ± SD (*n* = 3 for G2-NH_2_ and G4-NH_2_; *n* = 4 for G4-OH; *n* = 6 for G3.5-COOH). No statistically significant differences were observed compared to the vehicle control.

We applied oscillatory rheometry to measure the key viscoelastic parameters of the fibrin matrix, namely the storage modulus (*G*′), reflecting elasticity/stiffness (reversible deformation), and the loss modulus (*G*′’), reflecting viscosity/plasticity (irreversible deformation).^67^ Moreover, we also obtained the loss tangent, defined as tan δ = *G*′’/*G*′, which represents the relative plastic component during deformation. For these experiments, human plasma was incubated with dendrimers and treated with thrombin and Ca^2+^ to initiate clotting. Figure 9 shows average curves of the *G*′ and *G*′’ moduli measured during the polymerization process (Figure 9A), alongside the average measured values of *G*′, *G*′’, and tan δ (Figure 9B). Although some trends in *G*′ changes caused by dendrimers were observed relative to the control, these differences were not statistically significant.

### 3.8 Plasma Clot Fibrinolysis

Fibrinolysis refers to the slow breakdown of a blood clot to restore normal blood flow.^68,69^ Dendrimers could disrupt normal fibrinolysis through different mechanisms, including direct binding and inhibition of key fibrinolytic proteins or altering the ultrastructure and overall architecture of fibrin clots. For instance, in purified systems, anionic dendrimers have been reported to significantly inhibit plasminogen activation by tPA, though this effect was observed at much higher dendrimer concentrations than those used here.^37^ Understanding the impact of dendrimers on fibrinolysis is important, since enhanced fibrinolysis is associated with an increased tendency for bleeding and delayed or compromised wound healing, whereas impaired fibrinolysis is linked to the development of thrombosis.

**Figure 10:**
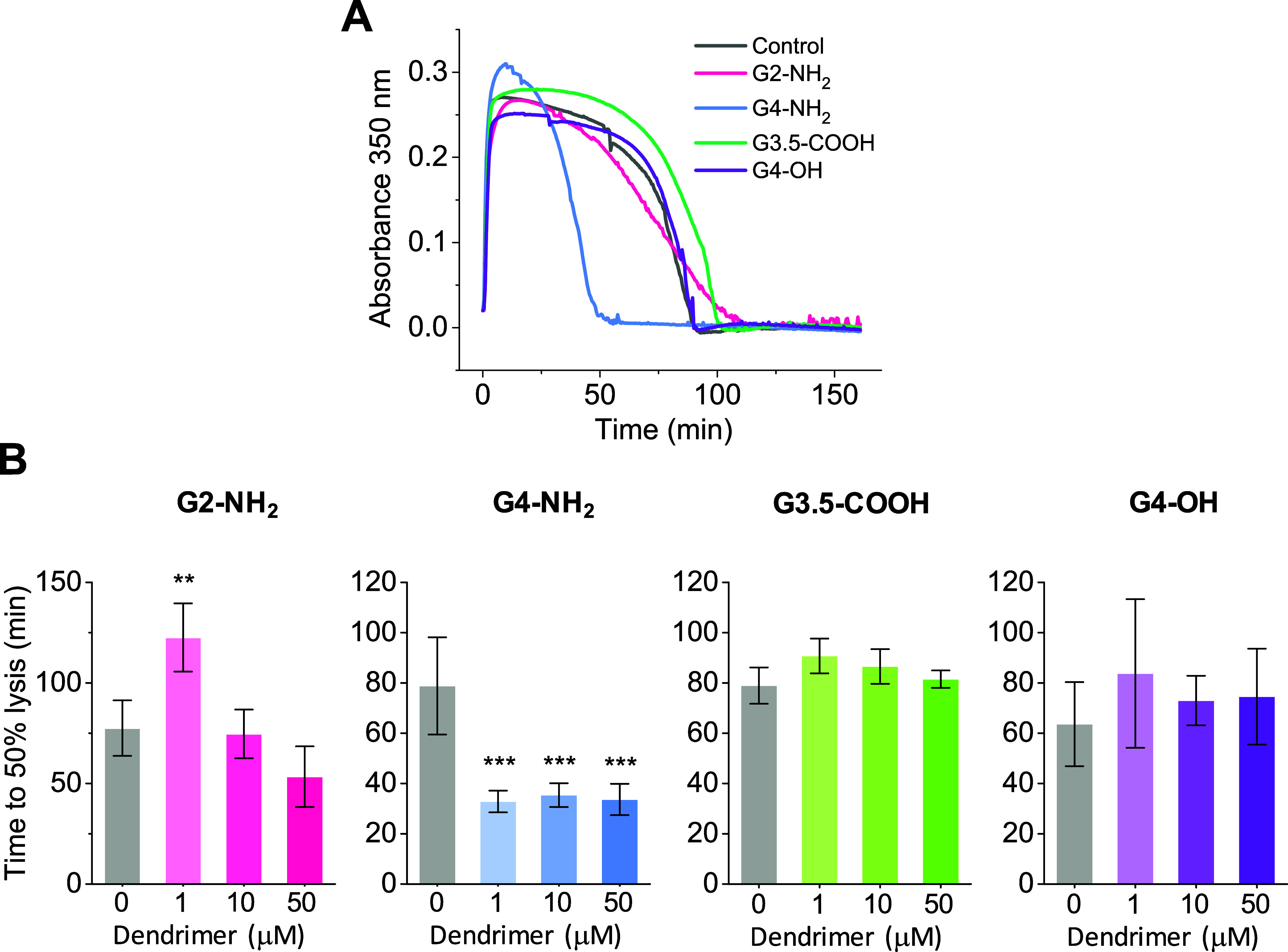
Influence of dendrimers on clot lysis evaluated through optical turbidimetry. Citrated human plasma was diluted 1:3 in HBS and incubated with dendrimers at the indicated concentrations for 30 min. This was followed by the addition of thrombin (0.4 NIH/mL) and Ca^2+^ (10 mM) to trigger clotting. (A) Progress curves of fibrin clot formation and lysis for a dendrimer concentration of 10 μM. Curves represent the average of 5 measurements. (B) Time to 50% lysis determined from progress curves. Data are reported as mean ± SD (*n* = 4–6), with **p < 0.01 and ***p < 0.001 relative to the vehicle control.

To investigate the impact of dendrimers on fibrinolysis, we incubated human plasma with dendrimers and tissue plasminogen activator (tPA). Next, we added thrombin and Ca^2+^ to initiate clotting, and then monitored clot formation and lysis through optical turbidimetry. The addition of tPA accelerates lysis by speeding up the conversion of plasminogen into plasmin. The results revealed that G4-NH_2_ significantly accelerated lysis (Figure 10), which is likely due to alterations in the overall fibrin network structure (Figure 7). Apart from a slight increase in lysis time induced by G2-NH_2_ at 1 μM, no additional changes in the dynamics of fibrinolysis were observed.

### 3.9 Hemolysis and Platelet Aggregation

We evaluated the potential impact of dendrimers on RBCs and platelets before proceeding with studies using whole blood (Section 3.10). Furthermore, platelets and RBCs are key components of the blood clotting process. Platelets aggregate to form a plug following vascular injury, while also serving as a scaffold to support and accelerate the fibrinogen coagulation process.^21^ RBCs, in turn, interact with fibrinogen, are incorporated into whole blood clots, and affect the structure and mechanical properties of these clots.^70^

We found that G4-NH_2_ caused a slight increase in percent hemolysis at 50 μM, while the other dendrimers had no effect (Supporting Figure S6). To study the impact of dendrimers on platelet aggregation, we used light transmission aggregometry with platelet-rich human plasma. The results indicated that G4-NH_2_ at 50 μM produced some platelet aggregation on its own, while the other dendrimers showed no effect (Supporting Figure S7A). Moreover, the results revealed that none of the dendrimers inhibited platelet aggregation when this was stimulated by the agonist arachidonic acid (Supporting Figure S7B).

### 3.10 Rotational Thromboelastometry in Whole Blood

We used the ROTEM technique to determine the influence of dendrimers on the coagulation process in human whole blood. This method measures changes in the viscoelastic properties of blood during coagulation.^71,72^ Key parameters extracted from this technique include: (i) CT, which measures the time from the onset of activation until the start of coagulation; (ii) clot formation time (CFT), which is the time from CT until a clot amplitude of 20 mm, reflecting the speed of clot strengthening and consolidation; (iii) clot polymerization rate (α-angle), which gauges the early rate of fibrin polymerization; (iv) maximum clot firmness (MCF), which measures clot strength in millimeters and reflects the combined effect of platelets, fibrinogen, and other clotting factors; (v) clot lysis index at 30/45/60 min (LI30/45/60), defined as the percentage of MCF remaining at the given time, used to evaluate the extent of clot breakdown or fibrinolysis.

**Figure 11:**
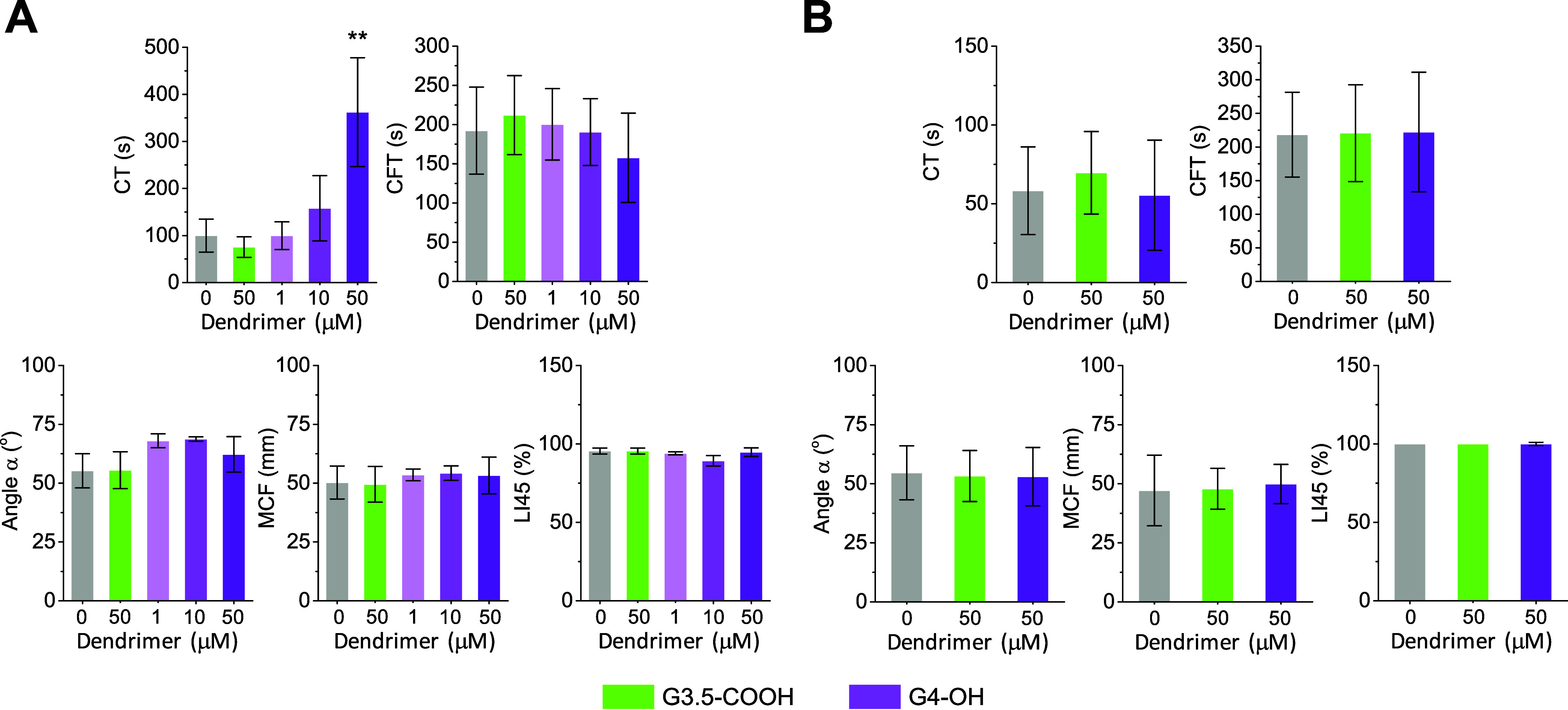
ROTEM characterization of the coagulation process in human whole blood. Whole blood was incubated with dendrimers at the indicated concentrations for 30 min, followed by the addition of Actin FS with Ca^2+^ (10 mM) or Thromborel S with Ca^2+^ (10 mM) to trigger clotting. (A,B) Characteristic ROTEM parameters obtained from whole blood samples activated with (A) Actin FS or (B) Thromborel S. Data are reported as mean ± SD (*n* = 3–4 in A; *n* = 6–8 in B), with **p < 0.01 relative to vehicle control.

We characterized the effects of G3.5-COOH and G4-OH in blood samples treated with Actin FS. We excluded the cationic dendrimers from this analysis because they have already been shown to significantly impair clot formation kinetics (Figures 3 and 4). The results showed that G3.5-COOH did not alter any of the ROTEM parameters compared to control measurements (Figure 11). Similarly, G4-OH did not affect the CFT, α-angle, MCF, or LI45 parameters. However, G4-OH significantly prolonged CT at 50 μM, but not at lower concentrations. When the coagulation cascade was triggered at the level of TF using Thromborel S, neither G3.5-COOH nor G4-OH altered any of the ROTEM parameters.

Interestingly, the ROTEM investigations in whole blood were consistent with the aPTT and PT assay findings, which indicated that G3.5-COOH had no effect on CT, while G4-OH at 50 μM prolonged CT in the aPTT but not in the PT assay (Figure 4). Results from the TGA assay also aligned with this overall picture, showing that G4-OH delayed TG when the coagulation cascade was triggered at the level of FXII (Figure 5A).

## 4 Conclusions

We investigated the effects of low-generation PAMAM dendrimers on the kinetics of fibrin clot formation, as well as the structure, properties, and stability of these clots. For this purpose, we employed a variety of analytical techniques and methodologies to gather comprehensive information on the clotting process and resulting fibrin clots. This multifaceted approach is crucial because nanomaterials can perturb various stages of the coagulation pathway. On the other hand, relying on a single diagnostic test provides limited insights and an incomplete assessment of the potential harmful effects of nanomaterials.

Our findings are summarized in Table 1. They indicate that all dendrimers influenced at least one clotting parameter. Overall, both cationic dendrimers appeared to be unsuitable for in vivo applications, particularly the larger G4-NH_2_. In comparison, G3.5-COOH and G4-OH showed much less impact on the clotting process. However, for clinically relevant G4-OH dendrimers, we obtained a surprising result, in that G4-OH prolonged clotting time not only in human plasma but also in whole blood, although this occurred only at high dendrimer concentrations (50 μM). Interestingly, G3.5-COOH showed potential as a safer option with respect to the clotting process, since they induced minimal alterations across most tested metrics.

Dissecting the molecular mechanisms underlying the effects described in Table 1 is a complex challenge, as dendrimers may interfere with multiple proteins across the contact, coagulation, and fibrinolytic systems. Nevertheless, it is useful to highlight a few particular aspects related to our findings. (i) For G2-NH_2_, G4-NH_2_, and G4-OH, we found that the prolonged clotting time in human plasma was due to impaired TG, although the mechanism behind this impairment was not investigated. This result is particularly interesting as it contrasts sharply with findings for similarly sized anionic usGNPs, which prolonged clotting time not by interfering with TG but by directly binding to fibrinogen.^49^ (ii) We found that G4-NH_2_ induced fibrin aggregation in human plasma. This probably accounts for the increased clot permeability/clot porosity associated with these dendrimers. This fibrin aggregation also likely explains the significant reduction in clot lysis time, as the aggregated fibers create more space and facilitate plasmin diffusion within the fibrin mesh. While we also anticipated an impact on the viscoelastic properties of clots treated with G4-NH_2_, we did not observe significant changes (there was a trend toward reduced *G*′, but without statistical significance). In contrast, the other dendrimers showed no alterations in fibrin ultrastructural properties (diameter and number of protofibrils) or in the overall architecture of the fibrin mesh. This is in line with the observation that these dendrimers did not affect clot permeability or lysis time. (iii) It is noteworthy that both G3.5-COOH and G4-OH, particularly G3.5-COOH, caused a decrease in maximum turbidity in purified fibrinogen samples. Quantitative analysis revealed that this reduction correlated with a lower number of protofibrils per fiber. This finding suggests that even these more inert dendrimers can modulate aspects of clot architecture. However, in plasma, the presence of other competing dendrimer-protein interactions mitigates these effects, preventing noticeable changes in fibrin clot structure.

**Table 1:** Summary of Main Findings^a^

**parameter**	**G2-NH**_**2**_	**G4-NH**_**2**_	**G3.5-COOH**	**G4-OH**
**turbidimetry/fibrinogen**				
time to 5% clotting	↓	↓↓↓	↓↓↓	↓
maximum absorbance	↑	↑↑↑	↓↓↓	↓
**turbidimetry/plasma**				
lag time/thrombin as initiator	—	—	—	—
lag time/Actin FS as initiator	↑↑↑	↑↑↑	—	—
**aPTT**				
clotting time	↑↑↑	↑↑↑	—	↑
**PT**				
clotting time	↑	↑↑↑	—	—
**TGA/Actin FS as initiator**				
lag time	↑↑↑	↑↑↑	—	↑
time to peak	↑↑↑	↑↑↑	—	↑
peak thrombin concentration	↓↓↓	↓↓↓	—	—
area under the curve	↓↓↓	↓↓↓	—	—
**TGA/innovin as initiator**				
lag time	↑	—	—	—
time to peak	↑	—	↓	↓
peak thrombin concentration	↓	↓↓↓	↑	—
area under the curve	↓	↓↓↓	—	—
**fibrinolysis**				
time to 50% lysis	↑	↓↓↓	—	—
**clot ultrastructure/fibrinogen**				
fiber diameter	N/A	N/A	—	—
number of protofibrils per fiber	N/A	N/A	↓↓↓	↓
**clot ultrastructure/plasma**				
fiber diameter	N/A	N/A	—	—
number of protofibrils per fiber	N/A	N/A	—	—
**confocal microscopy**				
structural change	—	↑↑↑	—	—
**clot permeability**				
Darcy’s constant (*K*s)	—	↑	—	—
**clot viscoelasticity**				
storage modulus (*G*′)	—	—	—	—
loss modulus (*G*′’)	—	—	—	—
loss tangent	—	—	—	—
hemolysis				
percent hemolysis	—	↑	—	—
**platelet aggregation**				
aggregation initiation	—	↑	—	—
inhibition of aggregation	—	—	—	—
**ROTEM/actin FS as initiator**				
clotting time (CT)	N/A	N/A	—	↑
clot formation time (CFT)	N/A	N/A	—	—
α-angle	N/A	N/A	—	—
maximum clot firmness (MCF)	N/A	N/A	—	—
lysis index at 45 min (LI45)	N/A	N/A	—	—
**ROTEM/thromborel as initiator**				
clotting time (CT)	N/A	N/A	—	—
clot formation time (CFT)	N/A	N/A	—	—
α-angle	N/A	N/A	—	—
maximum clot firmness (MCF)	N/A	N/A	—	—
lysis index at 45 min (LI45)	N/A	N/A	—	—

a Arrows (↑ and ↓) indicate a mild increase or decrease, whereas multiple arrows (↑↑↑ and ↓↓↓) denote a significant increase or decrease in the measured clot parameter relative to control conditions. A significant increase/decrease is defined as a high-magnitude change and/or a parameter change occurring at all tested dendrimer concentrations. A dash (—) indicates no change in the clot parameter, while N/A signifies that the parameter was not analyzed.

In summary, our results highlight the potential risks and benefits associated with dendrimer use in clinical settings. More broadly, fundamental studies like this one can provide valuable insights into the inherent biological properties of dendrimers.^73^ Understanding how to harness these properties, including the modulatory effects of dendrimers within the contact, coagulation, and fibrinolytic systems, could open new avenues for clinical applications.

## Supporting Information Available

Influence of dendrimers on fibrin aggregation, FXII activation, thrombin activity, and plasma clot formation; Quantitative parameters derived from real-time thrombin generation curves; Influence of dendrimers on hemolysis and platelet aggregation (PDF)

## References

[ref1] Svenson S.; Tomalia D. A. Dendrimers in biomedical applications—reflections on the field. Adv. Drug Delivery Rev. 2012, 64, 102–115. 10.1016/j.addr.2012.09.030. 16305813

[ref2] Pedziwiatr-Werbicka E.; Milowska K.; Dzmitruk V.; Ionov M.; Shcharbin D.; Bryszewska M. Dendrimers and hyperbranched structures for biomedical applications. Eur. Polym. J. 2019, 119, 61–73. 10.1016/j.eurpolymj.2019.07.013.

[ref3] Dias A. P.; da Silva Santos S.; da Silva J. V.; Parise-Filho R.; Ferreira E. I.; El Seoud O.; Giarolla J. Dendrimers in the context of nanomedicine. Int. J. Pharm. 2020, 573, 118814 10.1016/j.ijpharm.2019.118814. 31759101

[ref4] Wu L.-p.; Ficker M.; Christensen J. B.; Trohopoulos P. N.; Moghimi S. M. Dendrimers in medicine: therapeutic concepts and pharmaceutical challenges. Bioconjugate Chem. 2015, 26 (7), 1198–1211. 10.1021/acs.bioconjchem.5b00031. 25654320

[ref5] Shcharbin D.; Zhogla V.; Abashkin V.; Gao Y.; Majoral J. P.; Mignani S.; Shen M.; Bryszewska M.; Shi X. Recent advances in multifunctional dendrimer-based complexes for cancer treatment. WIREs Nanomed. Nanobiotechnol. 2024, 16 (2), e1951 10.1002/wnan.1951. 38456205

[ref6] Araújo R. V. d.; Santos S. d. S.; Igne Ferreira E.; Giarolla J. New advances in general biomedical applications of PAMAM dendrimers. Molecules 2018, 23 (11), 2849 10.3390/molecules23112849. 30400134 PMC6278347

[ref7] Gupta L.; Sharma A. K.; Gothwal A.; Khan M. S.; Khinchi M. P.; Qayum A.; Singh S. K.; Gupta U. Dendrimer encapsulated and conjugated delivery of berberine: A novel approach mitigating toxicity and improving in vivo pharmacokinetics. Int. J. Pharm. 2017, 528 (1–2), 88–99. 10.1016/j.ijpharm.2017.04.073. 28533175

[ref8] Zhang S.; Lloveras V.; Lope-Piedrafita S.; Calero-Pérez P.; Wu S.; Candiota A. P.; Vidal-Gancedo J. Metal-free radical dendrimers as MRI contrast agents for glioblastoma diagnosis: Ex vivo and in vivo approaches. Biomacromolecules 2022, 23 (7), 2767–2777. 10.1021/acs.biomac.2c00088. 35749573 PMC9277593

[ref9] Kannan S.; Dai H.; Navath R. S.; Balakrishnan B.; Jyoti A.; Janisse J.; Romero R.; Kannan R. M. Dendrimer-based postnatal therapy for neuroinflammation and cerebral palsy in a rabbit model. Sci. Transl. Med. 2012, 4 (130), 130ra46 10.1126/scitranslmed.3003162. PMC3492056 22517883

[ref10] Caminade A.-M. Dendrimers, an emerging opportunity in personalized medicine?. J. Person. Med. 2022, 12 (8), 1334 10.3390/jpm12081334. PMC9409959 36013283

[ref11] Gusdon A. M.; Faraday N.; Aita J. S.; Kumar S.; Mehta I.; Choi H. A.; Cleland J. L.; Robinson K.; McCullough L. D.; Ng D. K.; Kannan R. M.; Kannan S. Dendrimer nanotherapy for severe COVID-19 attenuates inflammation and neurological injury markers and improves outcomes in a phase2a clinical trial. Sci. Transl. Med. 2022, 14 (654), eabo2652 10.1126/scitranslmed.abo2652. 35857827

[ref12] Patterson C. M.; Balachander S. B.; Grant I.; Pop-Damkov P.; Kelly B.; McCoull W.; Parker J.; Giannis M.; Hill K. J.; Gibbons F. D.; et al. Design and optimization of dendrimer-conjugated Bcl-2/xL inhibitor, AZD0466, with improved therapeutic index for cancer therapy. Comm. Biol. 2021, 4 (1), 112 10.1038/s42003-020-01631-8. PMC7835349 33495510

[ref13] Sharma R.; Sharma A.; Kambhampati S. P.; Reddy R. R.; Zhang Z.; Cleland J. L.; Kannan S.; Kannan R. M. Scalable synthesis and validation of PAMAM dendrimer-N-acetyl cysteine conjugate for potential translation. Bioeng. Transl. Med. 2018, 3 (2), 87–101. 10.1002/btm2.10094. 30065965 PMC6063872

[ref14] Sonzini S.; Caputo F.; Mehn D.; Calzolai L.; Borgos S. E.; Hyldbakk A.; Treacher K.; Li W.; Jackman M.; Mahmoudi N.; Lawrence M. J.; Patterson C.; Owen D.; Ashford M.; Akhta N. In depth characterization of physicochemical critical quality attributes of a clinical drug-dendrimer conjugate. Int. J. Pharm. 2023, 637, 122905 10.1016/j.ijpharm.2023.122905. 37003312 PMC10157317

[ref15] Chis A. A.; Dobrea C.; Morgovan C.; Arseniu A. M.; Rus L. L.; Butuca A.; Juncan A. M.; Totan M.; Vonica-Tincu A. L.; Cormos G.; et al. Applications and limitations of dendrimers in biomedicine. Molecules 2020, 25 (17), 3982 10.3390/molecules25173982. 32882920 PMC7504821

[ref16] Shcharbin D.; Shcharbina N.; Dzmitruk V.; Pedziwiatr-Werbicka E.; Ionov M.; Mignani S.; de la Mata F. J.; Gomez R.; Muñoz-Fernández M. A.; Majoral J.-P.; Bryszewska M. Dendrimer-protein interactions versus dendrimer-based nanomedicine. Colloids Surf., B 2017, 152, 414–422. 10.1016/j.colsurfb.2017.01.041. 28167455

[ref17] Shcharbin D.; Janaszewska A.; Klajnert-Maculewicz B.; Ziemba B.; Dzmitruk V.; Halets I.; Loznikova S.; Shcharbina N.; Milowska K.; Ionov M.; et al. How to study dendrimers and dendriplexes III. Biodistribution, pharmacokinetics and toxicity in vivo. J. Controlled Release 2014, 181, 40–52. 10.1016/j.jconrel.2014.02.021. 24607663

[ref18] Dobrovolskaia M. Dendrimers effects on the immune system: insights into toxicity and therapeutic utility. Curr. Pharm. Des. 2017, 23 (21), 3134–3141. 10.2174/1381612823666170309151958. 28294045

[ref19] Li X.; Naeem A.; Xiao S.; Hu L.; Zhang J.; Zheng Q. Safety challenges and application strategies for the use of dendrimers in medicine. Pharmaceutics 2022, 14 (6), 1292 10.3390/pharmaceutics14061292. 35745863 PMC9230513

[ref20] Saha A. K.; Zhen M.-Y. S.; Erogbogbo F.; Ramasubramanian A. K. Design Considerations and Assays For Hemocompatibility of FDA-approved Nanoparticles, Sem. Thromb. Hemost.; Thieme Medical Publishers, 2020; pp 637–652. 10.1055/s-0039-1688491 31404934

[ref21] Brummel-Ziedins K.; Mann K. G. Molecular basis of blood coagulation. In Hematology; Elsevier, 2018; pp 1885–1905. e8.

[ref22] Tran H. D. N.; Moonshi S. S.; Xu Z. P.; Ta H. T. Influence of nanoparticles on the haemostatic balance: between thrombosis and haemorrhage. Biomater. Sci. 2021, 10 (1), 10–50. 10.1039/D1BM01351C. 34775503

[ref23] Kizhakkedathu J. N.; Conway E. M. Biomaterial and cellular implants: foreign surfaces where immunity and coagulation meet. Blood 2022, 139 (13), 1987–1998. 10.1182/blood.2020007209. 34415324

[ref24] Simak J.; De Paoli S. The effects of nanomaterials on blood coagulation in hemostasis and thrombosis. WIREs Nanomed. Nanobiotechnol. 2017, 9 (5), e1448 10.1002/wnan.1448. 28078811

[ref25] Konrath S.; Mailer R. K.; Renné T. Mechanism, functions, and diagnostic relevance of FXII activation by foreign surfaces. Hämostaseologie 2021, 41 (06), 489–501. 10.1055/a-1528-0499. 34592776

[ref26] Feller T.; Connell S. D.; Ariëns R. A. Why fibrin biomechanical properties matter for hemostasis and thrombosis. J. Thromb. Haemostasis 2022, 20 (1), 6–16. 10.1111/jth.15531. 34528378

[ref27] Litvinov R. I.; Pieters M.; de Lange-Loots Z.; Weisel J. W. Fibrinogen Fibrin 2021, 96, 471–501. 10.1007/978-3-030-58971-4_15. 33252741

[ref28] Zakharov A.; Awan M.; Gopinath A.; Lee S.-J. J.; Ramasubramanian A. K.; Dasbiswas K. Clots reveal anomalous elastic behavior of fiber networks. Sci. Adv. 2024, 10 (2), eadh1265 10.1126/sciadv.adh1265. 38198546 PMC10780871

[ref29] Dobrovolskaia M. A.; Patri A. K.; Potter T. M.; Rodriguez J. C.; Hall J. B.; McNeil S. E. Dendrimer-induced leukocyte procoagulant activity depends on particle size and surface charge. Nanomedicine 2012, 7 (2), 245–256. 10.2217/nnm.11.105. 21957862

[ref30] Jones C. F.; Campbell R. A.; Franks Z.; Gibson C. C.; Thiagarajan G.; Vieira-de-Abreu A.; Sukavaneshvar S.; Mohammad S. F.; Li D. Y.; Ghandehari H.; Weyrich A.; Brooks B. D.; Grainger D. W. Cationic PAMAM dendrimers disrupt key platelet functions. Mol. Pharmaceutics 2012, 9 (6), 1599–1611. 10.1021/mp2006054. PMC3367133 22497592

[ref31] Dobrovolskaia M. A.; Patri A. K.; Simak J.; Hall J. B.; Semberova J.; De Paoli Lacerda S. H.; McNeil S. E. Nanoparticle size and surface charge determine effects of PAMAM dendrimers on human platelets in vitro. Mol. Pharmaceutics 2012, 9 (3), 382–393. 10.1021/mp200463e. PMC3624701 22026635

[ref32] Watala C.; Karolczak K.; Kassassir H.; Talar M.; Przygodzki T.; Maczynska K.; Labieniec-Watala M. How do the full-generation poly (amido) amine (PAMAM) dendrimers activate blood platelets? Activation of circulating platelets and formation of “fibrinogen aggregates” in the presence of polycations. Int. J. Pharm. 2016, 503 (1–2), 247–261. 10.1016/j.ijpharm.2015.08.073. 26319628

[ref33] Klajnert B.; Pikala S.; Bryszewska M. Haemolytic activity of polyamidoamine dendrimers and the protective role of human serum albumin. Proc. R. Soc. A 2010, 466 (2117), 1527–1534. 10.1098/rspa.2009.0050.

[ref34] Markowicz-Piasecka M.; Sadkowska A.; Podsiedlik M.; Mikiciuk-Olasik E.; Sikora J. Generation 2 (G2)–Generation 4 (G4) PAMAM dendrimers disrupt key plasma coagulation parameters. Toxicol. In Vitro 2019, 59, 87–99. 10.1016/j.tiv.2019.04.010. 30981695

[ref35] Markowicz-Piasecka M.; Łuczak E.; Chałubiński M.; Broncel M.; Mikiciuk-Olasik E.; Sikora J. Studies towards biocompatibility of PAMAM dendrimers–overall hemostasis potential and integrity of the human aortic endothelial barrier. Int. J. Pharm. 2014, 473 (1–2), 158–169. 10.1016/j.ijpharm.2014.07.002. 24998508

[ref36] Fu Y.; Hu R.; Li C.; Wang Q.; Liu Z.; Xue W. Effects of poly (amidoamine) dendrimers on the structure and function of key blood components. J. Bioact. Compat. Polym. 2014, 29 (2), 165–179. 10.1177/0883911514521921.

[ref37] Aisina R.; Mukhametova L.; Ivanova E. Influence cationic and anionic PAMAM dendrimers of low generation on selected hemostatic parameters in vitro. Mater. Sci. Eng., C 2020, 109, 110605 10.1016/j.msec.2019.110605. 32228918

[ref38] Jones C. F.; Campbell R. A.; Brooks A. E.; Assemi S.; Tadjiki S.; Thiagarajan G.; Mulcock C.; Weyrich A. S.; Brooks B. D.; Ghandehari H.; Grainger D. W. Cationic PAMAM dendrimers aggressively initiate blood clot formation. ACS Nano 2012, 6 (11), 9900–9910. 10.1021/nn303472r. 23062017 PMC3532938

[ref39] Li G.; Zhang Y.; Tang W.; Zheng J. Comprehensive investigation of in vitro hemocompatibility of surface modified polyamidoamine nanocarrier. Clin. Hemorheol. Microcirc. 2020, 74 (3), 267–279. 10.3233/CH-190641. 31476147

[ref40] Mihalko E.; Brown A. C. Clot Structure and Implications for Bleeding and Thrombosis, Sem. Thromb. Hemost., Thieme Medical Publishers, 2020; pp 096–104. 10.1055/s-0039-1696944 PMC7460717 31614389

[ref41] Wolberg A. S. Plasma and cellular contributions to fibrin network formation, structure and stability. Haemophilia 2010, 16, 7–12. 10.1111/j.1365-2516.2010.02253.x. 20586795

[ref42] Ząbczyk M.; Ariëns R. A.; Undas A. Fibrin clot properties in cardiovascular disease: from basic mechanisms to clinical practice. Cardiovasc. Res. 2023, 119 (1), 94–111. 10.1093/cvr/cvad017. 36662542 PMC10377755

[ref43] Gidley G. N.; Holle L. A.; Burthem J.; Bolton-Maggs P. H.; Lin F.-C.; Wolberg A. S. Abnormal plasma clot formation and fibrinolysis reveal bleeding tendency in patients with partial factor XI deficiency. Blood Adv. 2018, 2 (10), 1076–1088. 10.1182/bloodadvances.2017015123. 29760205 PMC5965046

[ref44] Hugenholtz G.; Macrae F.; Adelmeijer J.; Dulfer S.; Porte R.; Lisman T.; Ariëns R. Procoagulant changes in fibrin clot structure in patients with cirrhosis are associated with oxidative modifications of fibrinogen. J. Thromb. Haemostasis 2016, 14 (5), 1054–1066. 10.1111/jth.13278. 26833718

[ref45] Driever E. G.; Lisman T. Fibrin clot properties and thrombus composition in cirrhosis. Res. Pract. Thromb. Haemostasis 2023, 7 (1), 100055 10.1016/j.rpth.2023.100055. 36798901 PMC9925609

[ref46] Konieczynska M.; Fil K.; Bazanek M.; Undas A. Prolonged duration of type 2 diabetes is associated with increased thrombin generation, prothrombotic fibrin clot phenotype and impaired fibrinolysis. Thromb. Haemostasis 2014, 111 (04), 685–693. 10.1160/TH13-07-0566. 24306139

[ref47] Siniarski A.; Baker S. R.; Duval C.; Malinowski K. P.; Gajos G.; Nessler J.; Ariëns R. A. Quantitative analysis of clot density, fibrin fiber radius, and protofibril packing in acute phase myocardial infarction. Thromb. Res. 2021, 205, 110–119. 10.1016/j.thromres.2021.06.024. 34298252

[ref48] Ruhoff A. M.; Hong J. K.; Gao L.; Singh J.; Tran C.; Mackie G.; Waterhouse A. Biomaterial wettability affects fibrin clot structure and fibrinolysis. Adv. Healthcare Mater. 2021, 10 (20), 2100988 10.1002/adhm.202100988. 34423587

[ref49] Mina N.; Guido V. S.; Lima A. F.; Oliva M. L. V.; Sousa A. A. Ultrasmall Nanoparticles Bind to Fibrinogen and Impair Normal Clot Formation. Part. Part. Syst. Charact. 2024, 41 (4), 2300107 10.1002/ppsc.202300107.

[ref50] Lira A. L.; Mina N.; Bonturi C. R.; Nogueira R. S.; Torquato R. J.; Oliva M. L. V.; Sousa A. A. Anionic Ultrasmall gold nanoparticles bind to coagulation factors and disturb normal hemostatic balance. Chem. Res. Toxicol. 2022, 35 (9), 1558–1569. 10.1021/acs.chemrestox.2c00190. 36018252

[ref51] Lira A. L.; Ferreira R. S.; Oliva M. L. V.; Sousa A. A. Regulation of thrombin activity with ultrasmall nanoparticles: effects of surface chemistry. Langmuir 2020, 36 (27), 7991–8001. 10.1021/acs.langmuir.0c01352. 32590899

[ref52] Potter T. M.; Rodriguez J. C.; Neun B. W.; Ilinskaya A. N.; Cedrone E.; Dobrovolskaia M. A. In vitro assessment of nanoparticle effects on blood coagulation. In Characterization of Nanoparticles Intended for Drug Delivery; McNeil S. E., Ed.; Humana Press, 2018; Chapter 10, pp 103–124. 10.1007/978-1-4939-7352-1_10 29039097

[ref53] Longstaff C. Development of Shiny app tools to simplify and standardize the analysis of hemostasis assay data: communication from the SSC of the ISTH. J. Thromb. Haemostasis 2017, 15 (5), 1044–1046. 10.1111/jth.13656. 28304129

[ref54] Belcher H. A.; Guthold M.; Hudson N. E. What is the diameter of a fibrin fiber?. Res. Pract. Thromb. Haemostasis 2023, 7 (5), 100285 10.1016/j.rpth.2023.100285. 37601015 PMC10439396

[ref55] Belcher H. A.; Litwa K.; Guthold M.; Hudson N. E. The applicability of current turbidimetric approaches for analyzing fibrin fibers and other filamentous networks. Biomolecules 2022, 12 (6), 807 10.3390/biom12060807. 35740932 PMC9221518

[ref56] Domingues M. M.; Macrae F. L.; Duval C.; McPherson H. R.; Bridge K. I.; Ajjan R. A.; Ridger V. C.; Connell S. D.; Philippou H.; Ariëns R. A. Thrombin and fibrinogen γ′ impact clot structure by marked effects on intrafibrillar structure and protofibril packing. Blood 2016, 127 (4), 487–495. 10.1182/blood-2015-06-652214. 26608329

[ref57] Guido V. S.; Olivieri P. H. Jr; Brito M. L.; Prezoto B. C.; Martinez D. S.; Oliva M. L. V.; Sousa A. A. Stealth and Biocompatible Gold Nanoparticles through Surface Coating with a Zwitterionic Derivative of Glutathione. Langmuir 2024, 40, 12167–12178. 10.1021/acs.langmuir.4c01123. 38808371 PMC11171461

[ref58] Wygrecka M.; Birnhuber A.; Seeliger B.; Michalick L.; Pak O.; Schultz A.-S.; Schramm F.; Zacharias M.; Gorkiewicz G.; David S.; et al. Altered fibrin clot structure and dysregulated fibrinolysis contribute to thrombosis risk in severe COVID-19. Blood Adv. 2022, 6 (3), 1074–1087. 10.1182/bloodadvances.2021004816. 34861681 PMC8648369

[ref59] Farkas Á. Z.; Farkas V. J.; Szabó L.; Wacha A.; Bóta A.; Csehi L.; Kolev K.; Thelwell C. Structure, mechanical, and lytic stability of fibrin and plasma coagulum generated by Staphylocoagulase from Staphylococcus aureus. Front. Immunol. 2019, 10, 2967 10.3389/fimmu.2019.02967. 31921206 PMC6933771

[ref60] Gould T. J.; Vu T. T.; Stafford A. R.; Dwivedi D. J.; Kim P. Y.; Fox-Robichaud A. E.; Weitz J. I.; Liaw P. C. Cell-free DNA modulates clot structure and impairs fibrinolysis in sepsis. Arterioscler., Thromb., Vasc. Biol. 2015, 35 (12), 2544–2553. 10.1161/ATVBAHA.115.306035. 26494232

[ref61] Pan X.; Gong Y. Y.; Martinelli I.; Angelici L.; Favero C.; Bertazzi P. A.; Mannucci P. M.; Ariëns R. A.; Routledge M. N. Fibrin clot structure is affected by levels of particulate air pollution exposure in patients with venous thrombosis. Environ. Int. 2016, 92–93, 70–76. 10.1016/j.envint.2016.03.030. 27060417

[ref62] Winter W. E.; Flax S. D.; Harris N. S. Coagulation testing in the core laboratory. Lab. Med. 2017, 48 (4), 295–313. 10.1093/labmed/lmx050. 29126301

[ref63] Wolberg A. S. Thrombin generation and fibrin clot structure. Blood Rev. 2007, 21 (3), 131–142. 10.1016/j.blre.2006.11.001. 17208341

[ref64] Al Dieri R.; De Laat B.; Hemker H. C. Thrombin generation: what have we learned?. Blood Rev. 2012, 26 (5), 197–203. 10.1016/j.blre.2012.06.001. 22762893

[ref65] Depasse F.; Binder N. B.; Mueller J.; Wissel T.; Schwers S.; Germer M.; Hermes B.; Turecek P. L. Thrombin generation assays are versatile tools in blood coagulation analysis: A review of technical features, and applications from research to laboratory routine. J. Thromb. Haemostasis 2021, 19 (12), 2907–2917. 10.1111/jth.15529. 34525255 PMC9291770

[ref66] Woodhead J. L.; Nagaswami C.; Matsuda M.; Arocha-Pinango C. L.; Weisel J. W. The Ultrastructure of Fibrinogen Caracas II Molecules, Fibers, and Clots (*). J. Biol. Chem. 1996, 271 (9), 4946–4953. 10.1074/jbc.271.9.4946. 8617768

[ref67] Litvinov R. I.; Weisel J. W. Fibrin mechanical properties and their structural origins. Matrix Biol. 2017, 60-61, 110–123. 10.1016/j.matbio.2016.08.003. 27553509 PMC5318294

[ref68] Longstaff C.; Kolev K. Basic mechanisms and regulation of fibrinolysis. J. Thromb. Haemostasis 2015, 13, S98–S105. 10.1111/jth.12935. 26149056

[ref69] Longstaff C. Measuring fibrinolysis. Hämostaseologie 2021, 41 (01), 069–075. 10.1055/a-1325-0268. 33588458

[ref70] Litvinov R. I.; Weisel J. W. Role of red blood cells in haemostasis and thrombosis. ISBT Sci. Ser. 2017, 12 (1), 176–183. 10.1111/voxs.12331. 28458720 PMC5404239

[ref71] Drotarova M.; Zolkova J.; Belakova K. M.; Brunclikova M.; Skornova I.; Stasko J.; Simurda T. Basic Principles of Rotational Thromboelastometry (ROTEM) and the Role of ROTEM—Guided Fibrinogen Replacement Therapy in the Management of Coagulopathies. Diagnostics 2023, 13 (20), 3219 10.3390/diagnostics13203219. 37892040 PMC10606358

[ref72] Zeng Z.; Fagnon M.; Chakravarthula T. N.; Alves N. J. Fibrin clot formation under diverse clotting conditions: Comparing turbidimetry and thromboelastography. Thromb. Res. 2020, 187, 48–55. 10.1016/j.thromres.2020.01.001. 31954276

[ref73] Kheraldine H.; Rachid O.; Habib A. M.; Al Moustafa A.-E.; Benter I. F.; Akhtar S. Emerging innate biological properties of nano-drug delivery systems: A focus on PAMAM dendrimers and their clinical potential. Adv. Drug Delivery Rev. 2021, 178, 113908 10.1016/j.addr.2021.113908. 34390777

