# Supplementary material for: How Dendrimers Impact Fibrin Clot Formation, Structure, and Properties
Source: ACS Omega. 2024 Dec 18;9(52):51306–19. doi: 10.1021/acsomega.4c08120 (PMC11696396; doi:10.1021/acsomega.4c08120)
Supplement: Supplementary file 1 — ao4c08120_si_001.pdf [file ao4c08120_si_001.pdf]

Supporting Information

How Dendrimers Impact Fibrin Clot Formation, Structure, and Properties

Natasha Mina,[1] Vinicius S. Guido,[1] Benedito C. Prezoto,[2] Maria Luiza V. Oliva,[1] Alioscka A. Sousa[1,*]

1. Department of Biochemistry, Federal University of São Paulo, São Paulo, SP 04044-020, Brazil

2. Laboratory of Pharmacology, Butantan Institute, São Paulo, SP 05503-900, Brazil

* alioscka.sousa@unifesp.br

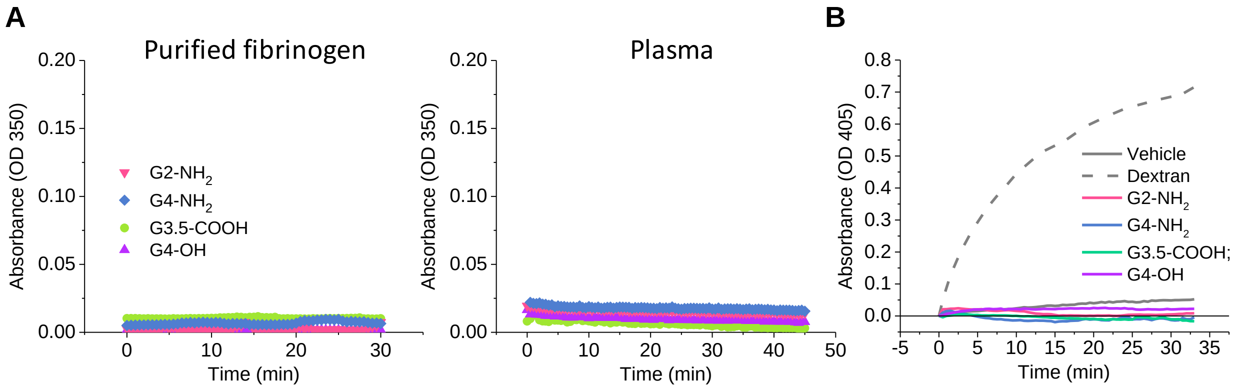

Figure S1. Control experiments demonstrating that the dendrimers themselves did not produce discernible changes in anticoagulated (citrated) human plasma. (A) Dendrimer-induced aggregation in samples of purified fibrinogen and human plasma. Dendrimers (50 μM) were incubated with samples of purified fibrinogen (4 μM) and human plasma (1:3 dilution in TBS), and absorbance readings were monitored over time at 350 nm. The results show that the tested dendrimers alone do not induce fibrinogen aggregation under the given conditions. (B) FXII and PK activation. Citrated human plasma was incubated with dendrimers (50 μM) for 15 min, followed by the addition of the chromogenic substrate S-2302 (300 μM). Amidolytic activities were recorded at 405 nm using a plate reader. Dextran sulfate (5 nM) was used as a positive control, while buffer served as the vehicle control. The results suggest that dendrimers do not activate the contact pathway in human plasma.

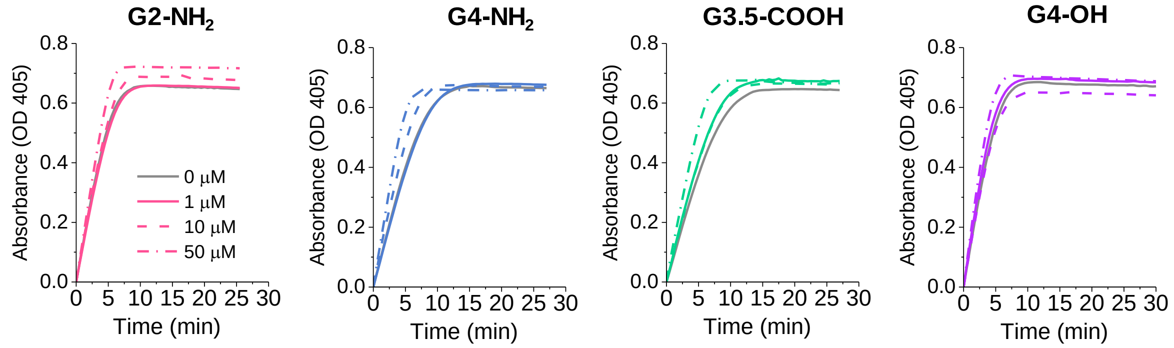

Figure S2. Influence of dendrimers on thrombin activity against a chromogenic substrate. Thrombin (0.4 NIH/mL) was incubated with dendrimers at the indicated concentrations for 15 min, followed by the addition of the chromogenic substrate S-2238 (300 μM). Amidolytic activities were recorded at 405 nm using a plate reader. The results suggest that changes in enzyme activity in the presence of dendrimers do not account for the trends in lag time observed in Figure 2. The legend under G2-NH2 applies to all panels.

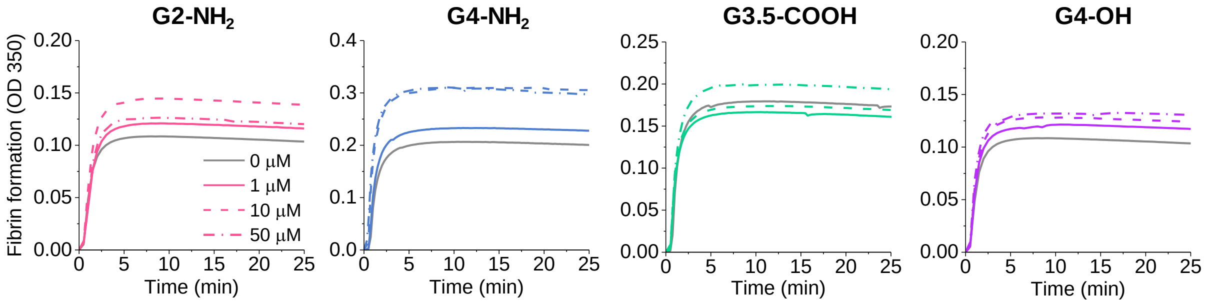

Figure S3. Influence of dendrimers on plasma clot formation studied by optical turbidimetry. Citrated human plasma was incubated with dendrimers at the indicated concentrations for 15 min, followed by the addition of thrombin to trigger clotting. The legend under G2-NH2 applies to all panels.

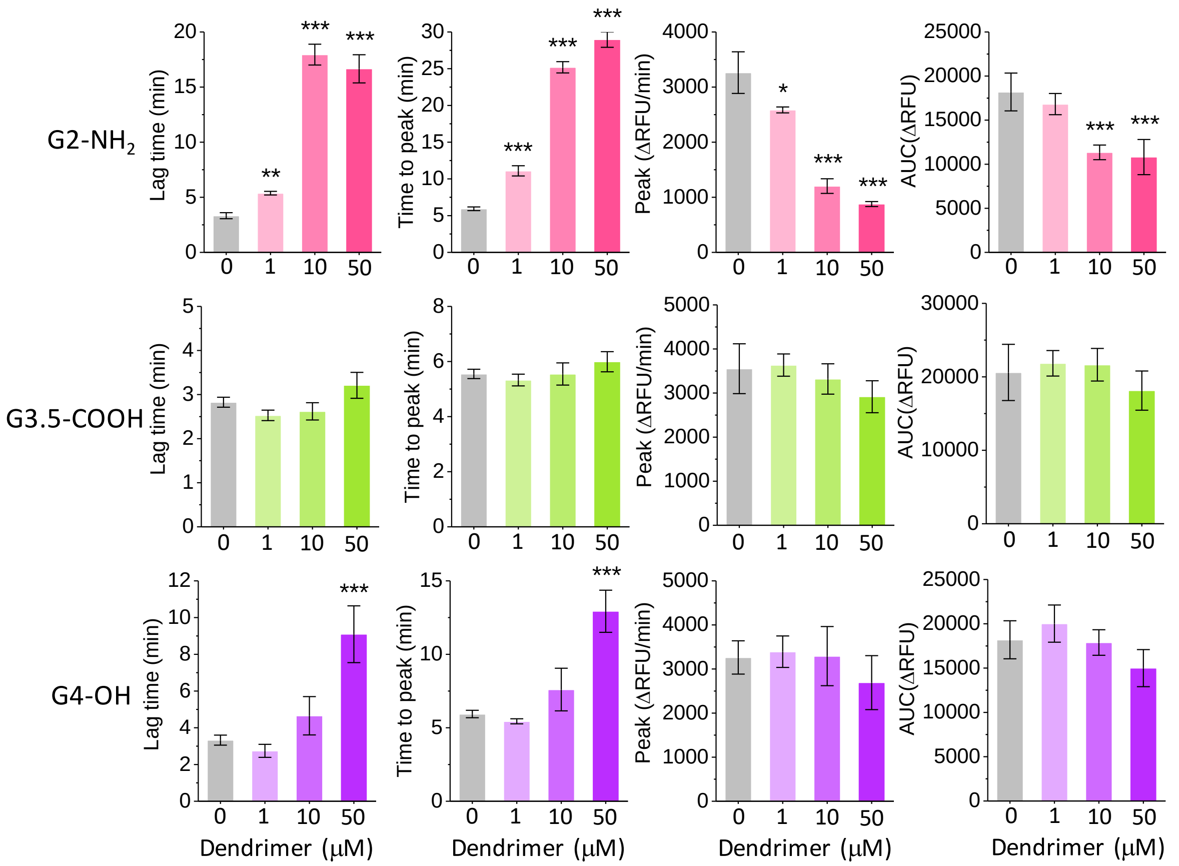

Figure S4. Influence of dendrimers on real-time thrombin generation in human plasma. Citrated human plasma was incubated with dendrimers for 30 min at the indicated concentrations, and Actin FS with Ca^{2+} (10 mM) was used to trigger clot formation. Shown are thrombin generation parameters derived from corresponding TGA curves (see Figure 5A for representative TGA curves). No thrombin generation was detected

in plasma incubated with G4-NH2. Data are reported as mean ± SD (n = 3−6), with *p < 0.05, **p < 0.01 and ***p < 0.001 relative to vehicle control.

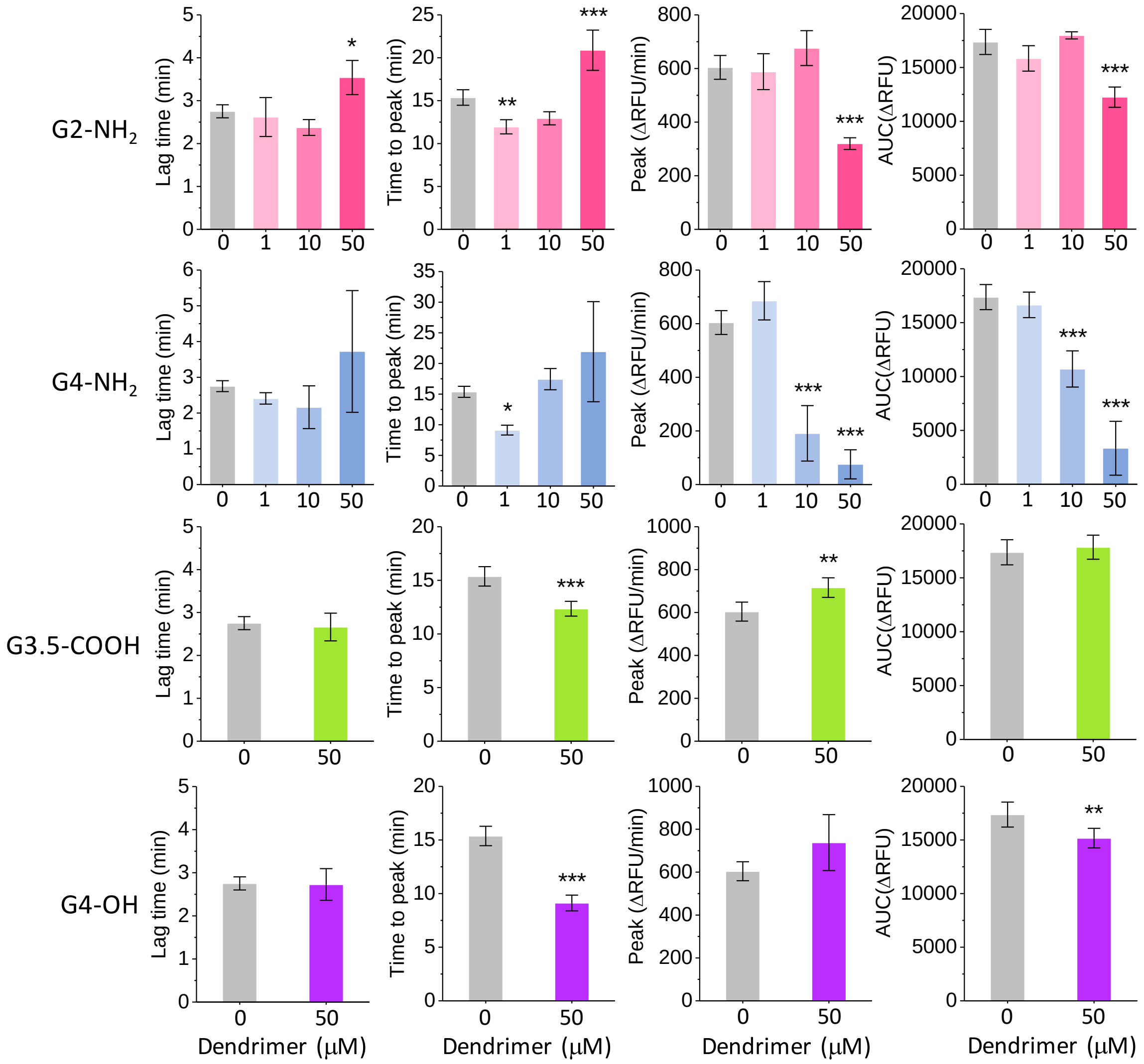

Figure S5. Influence of dendrimers on real-time thrombin generation in human plasma. Citrated human plasma was incubated with dendrimers for 30 min at the indicated concentrations, and Innovin with Ca^{2+} (10 mM) was used to trigger clot formation. Shown are thrombin generation parameters derived from corresponding TGA curves (see Figure 5B for representative TGA curves). Data are reported as mean ± SD (n = 5), with *p < 0.05, **p < 0.01, ***p < 0.001 relative to vehicle control.

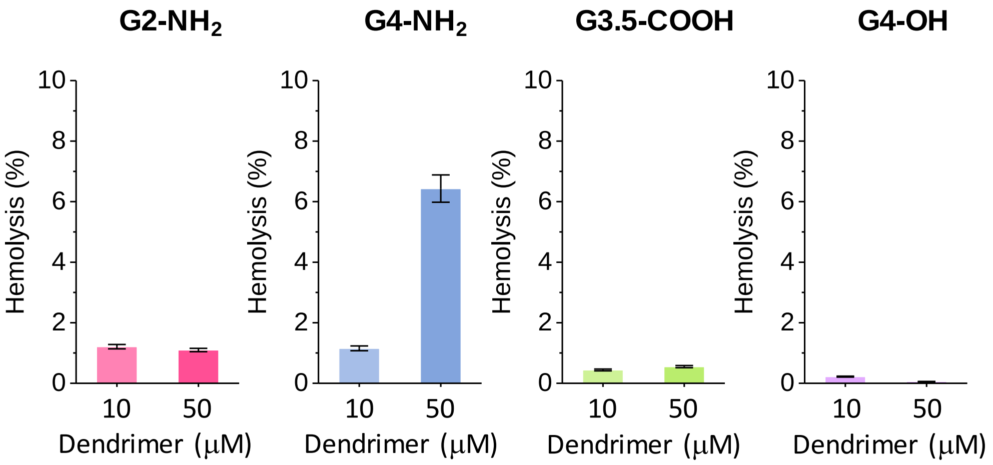

Figure S6. Influence of dendrimers on hemolysis. Whole blood diluted 1:50 in HBS was incubated with or without dendrimers (10 and 50 µM) for 4 h at 37 °C in an orbital shaker. Positive controls contained 1% Triton X-100. Following incubation, samples were centrifuged at 1500 g for 5 min, and the supernatants were collected. The supernatants were analyzed in a microplate reader with absorbance readings at 540 nm. Percent hemolysis was calculated according to: Hemolysis (%) = 100 × [(OD_{sample} – OD_{neg})/(OD_{pos}-OD_{neg})].

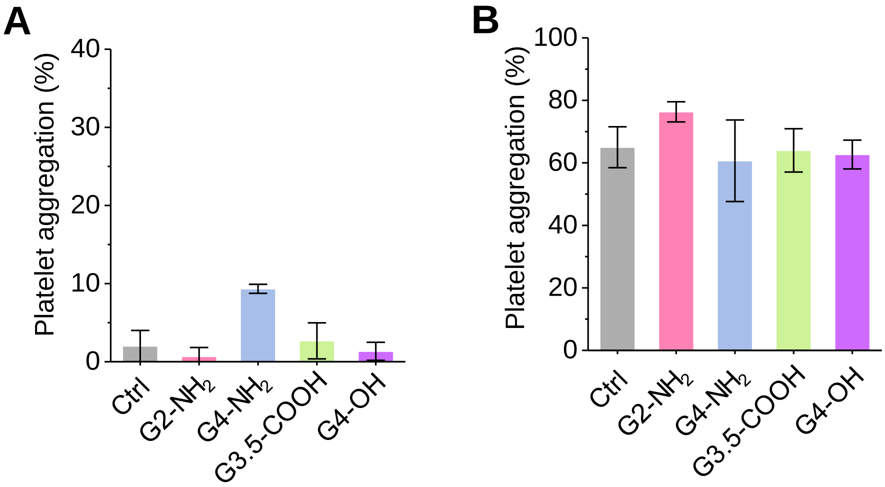

Figure S7. Influence of dendrimers on platelet aggregation. Suspensions of platelet-rich plasma (PRP) were incubated with or without dendrimers (50 µM) for 30 min at 37 °C. Subsequently, arachidonic acid (0.5 mM) was added to induce platelet aggregation, and samples were further incubated for 10 min. Platelet aggregation was monitored at 37 °C using a Chrono−Log 490 aggregometer (Chrono−Log Corporation). The degree of platelet aggregation was expressed as the percent change in light transmittance relative to PRP (0% light transmission) and buffer (100% light transmission). (A) Results indicate that G4-NH_2 initiated platelet aggregation on is own, while the other dendrimers showed on effect. (B) Results show that none of the dendrimers inhibited platelet aggregation when this was stimulated by arachidonic acid.
